# Proteomic discovery of DEK and NUMA1 as new players in UV-induced DNA damage repair mechanisms

**DOI:** 10.1038/s41420-025-02823-z

**Published:** 2025-11-24

**Authors:** Namwoo Kim, Mihyun Kim, Eunwoo Jeong, Jung-Eun Yeo, Byung-gyu Kim, Kyungjae Myung, Orlando D. Schärer, Kyoo-young Lee

**Affiliations:** 1 Center for Genomic Integrity, Institute for Basic Science, Ulsan, 44919 Korea; 2 Department of Biological Sciences, College of Information-Bio Convergence Engineering, Ulsan National Institute of Science and Technology, Ulsan, 44919 Korea; 3 Department of Biomedical Engineering, College of Information-Bio Convergence Engineering, Ulsan National Institute of Science and Technology, Ulsan, 44919 Korea; 4 Graduate School of Health Science and Technology, College of Information-Bio Convergence Engineering, Ulsan National Institute of Science and Technology, Ulsan, 44919 Korea; 5 UPMC Hillman Cancer Center & Department of Pharmacology and Chemical Biology, University of Pittsburgh School of Medicine, Pittsburgh, PA USA; 6 Department of Biochemistry, College of Medicine, Hallym University, Chuncheon, 24252 Korea

**Keywords:** Nucleotide excision repair, DNA metabolism

## Abstract

Ultraviolet (UV)-induced DNA lesions threaten genomic stability and are associated with skin carcinogenesis. These lesions are primarily repaired by the nucleotide excision repair (NER) pathway. However, alternative repair mechanisms and regulators are emerging as critical contributors to managing UV lesions. Here, we used a click chemistry-based proteomic approach to identify DEK and NUMA1 as novel regulators of UV-induced DNA lesion repair. Depletion of DEK or NUMA1 resulted in delayed UV lesion repair and increased cellular UV sensitivity. This was accompanied by delayed recruitment of XPF to UV-damaged sites. Notably, abnormal accumulation of proliferating cell nuclear antigen (PCNA) at UV lesions was observed in DEK- or NUMA1-depleted cells. This PCNA accumulation was not entirely dependent on NER, as it also involved contributions from apurinic/apyrimidinic endonuclease 1 (APE1), a key protein in base excision repair (BER). Co-depletion experiments revealed an epistatic relationship between DEK or NUMA1 and APE1, but not with XPA, suggesting an impaired BER in DEK- or NUMA1-depleted cells, possibly due to excessive PCNA accumulation. Our findings suggest that DEK and NUMA1 facilitate efficient UV lesion removal by promoting proper NER activity and regulating APE1-mediated long-patch BER, highlighting the collaborative roles of NER and BER in UV lesion repair.

## Introduction

Ultraviolet (UV)-induced DNA lesions have been implicated in an increased risk of skin cancer, highlighting the importance of efficient DNA repair mechanisms in maintaining genomic stability. Prolonged or excessive exposure to UV-B or UV-C radiation can lead to the formation of harmful DNA lesions, such as cyclobutane pyrimidine dimers (CPDs) and pyrimidine (6-4) pyrimidone photoproducts ((6-4)PPs), which are primarily repaired by the nucleotide excision repair (NER) pathway [1, 2]. Genetic defects in the NER pathway are associated with inherited diseases such as xeroderma pigmentosum (XP), Cockayne syndrome (CS), and trichothiodystrophy (TTD) [3], highlighting the versatile repair activity of NER.

NER is divided into two sub-pathways based on damage recognition step: global-genome NER (GG-NER) and transcription-coupled NER (TC-NER) [2]. In GG-NER, XPC-RAD23B detects DNA damage and recruits the transcription factor II H (TFIIH). In TC-NER, a stalled RNA polymerase II at the damage site recruits TFIIH with the help of the CSB, CSA, UVSSA, ELOF1 and STK19 proteins. Both pathways converge to form a preincision complex where TFIIH unwinds the DNA and verifies the lesion, and XPA, replication protein A (RPA), XPG, and XPF join to form the full preincision complex. The ERCC1-XPF endonuclease makes cuts the DNA 5’ to the lesion, and nascent DNA synthesis mediated by the DNA polymerases δ, ε, and κ, fills the incision gap [4–6]. After a 3’ cut by the XPG endonuclease and subsequent nick sealing, repair is complete [7]. Recent evidence has pointed to the complexity of the NER mechanism; in addition to the core NER proteins, accessory factors including chromatin modifying proteins and post-translational modification regulate the NER process in the cellular environment [2, 8, 9].

While NER is the primary pathway for the removal of UV-induced DNA lesions, recent studies have provided evidence for alternative DNA repair mechanisms. It has been reported that topoisomerase I (TOP1)-single strand breaks (SSB) complexes can be trapped by UV lesions, and that (6-4)PPs downstream of the SSB can be removed during repair synthesis in long-patch base excision repair (LP-BER) [10]. In addition, an APE1-dependent LP-BER pathway has been shown to mediate the repair of UV-induced DNA damage, especially in cells with defective NER activity [11]. While this broadens the scope of our understanding of how multiple repair pathways can contribute to the removal of UV-induced DNA lesions, the importance and coordination of these alternative pathways remains to be investigated.

To date, a majority of studies have focused on damage recognition and the assembly of the preincision complex in NER, while the postincision steps, including DNA repair synthesis, are less well understood. In this study, we used a click chemistry-based proteomic approach to identify proteins spatially associated with nascent DNA during NER repair synthesis following UV irradiation. Among the proteins isolated in this screen, were DEK proto-oncoprotein and Nuclear mitotic apparatus protein 1 (NUMA1), proteins known for their role in the regulation of transcription and spindle regulation, respectively [12, 13]. Both proteins have been reported to have roles in the regulation of DNA double-strand break repair [14–16]. Our study reveals that they play a role in the repair of UV-induced DNA lesions by influencing both NER and BER activities at UV-induced lesions. Our findings shed light on the cooperative role of multiple repair pathways in maintaining genomic stability in the face of UV irradiation-induced DNA damage.

## Results

### DEK and NUMA1 are involved in the repair of UV-induced DNA lesions

**Fig. 1:**
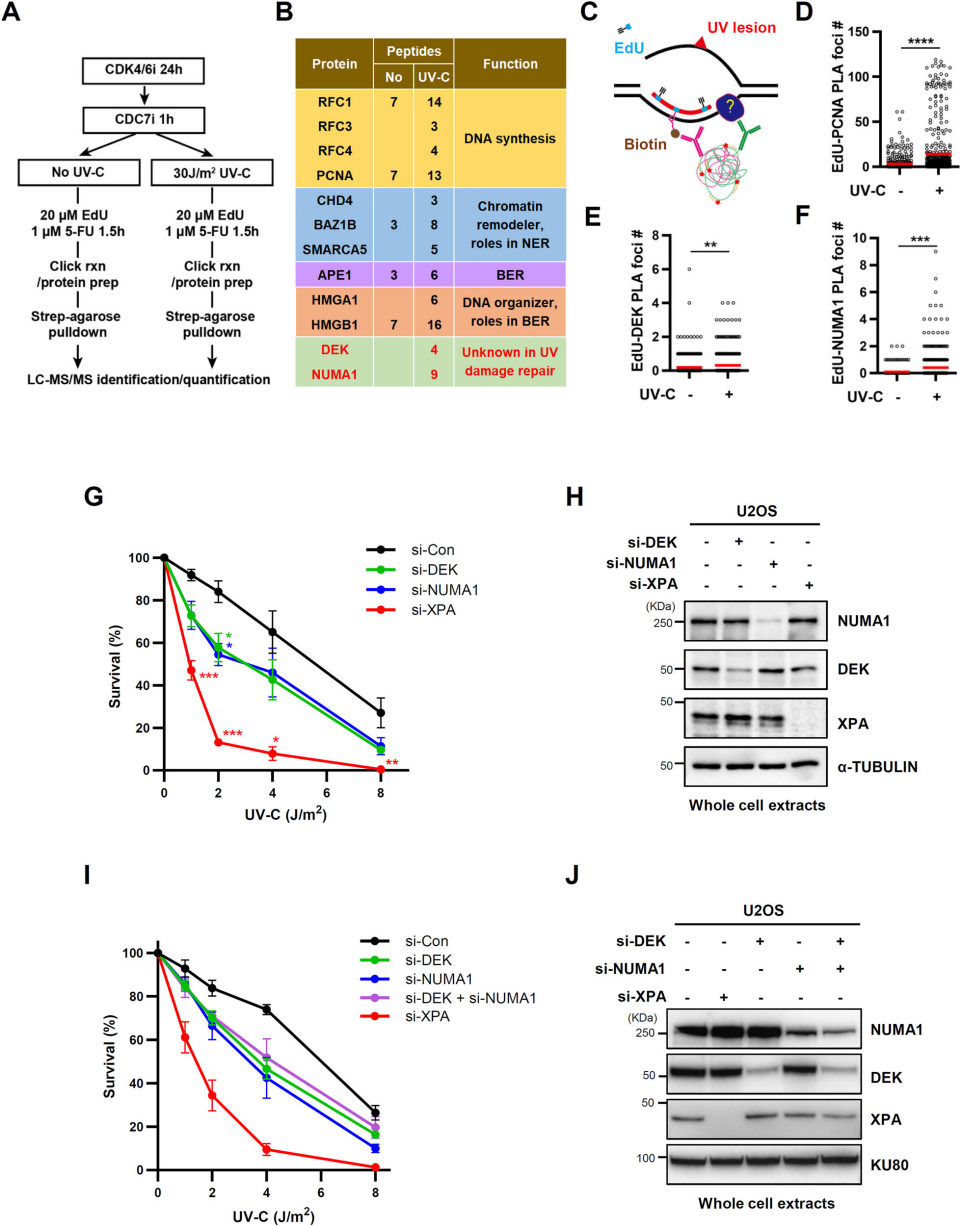
DEK and NUMA1 were identified as novel players in UV-induced DNA lesion repair. **A** The experimental scheme for the identification of proteins associated with UV-induced DNA synthesis. Detailed procedure was described in the method section. **B** A list of several proteins identified by the experimental scheme in **A**. The number of peptides detected is shown. **C–F** U2OS cells arrested in the G1 phase by treatment with CDK4/6i and CDC7i were irradiated with 30 J/m^2^ UV-C, and EdU was incorporated and fixed for a PLA assay between biotin-clicked EdU and PCNA **D**, DEK **E**, or NUMA1 **F**. **C** Graphic scheme of a PLA assay. **D–F** A representative graph of three biological replicates is shown. Red line indicates mean. **G–J** U2OS cells were transfected with siRNAs as indicated. 48 h after transfection, cells were irradiated with UV-C and subjected to clonogenic survival assay **G**, **I** or lysed for Western blot **H**, **J**. **G**, **I** Error bars represent standard error of the mean (SEM) (*n*> 3). Each colored asterisk represents the statistical analysis in comparison to si-Con. Statistical analysis: two-tailed unpaired Student’s *t*-test **D–F**; one-way ANOVA **G**, **I**. ***P < 0.005, **P < 0.01, *P < 0.05, and otherwise not significant.

To develop an unbiased approach to identify novel factors involved in the repair of UV-induced DNA lesions, we adapted iPOND (isolation of proteins on nascent DNA), an approach used to monitor protein dynamics at the replication fork [17], for use with repair synthesis following UV-induced DNA damage. To minimize interference from replication-associated nascent DNA synthesis, U2OS cells were arrested in G1 using the CDK4/6 inhibitor (CDK4/6i) palbociclib and origin firing was blocked by the CDC7 inhibitor (CDC7i) PHA767491 (Fig. 1A and Supplementary Fig. 1A). Cells were then irradiated with ultraviolet-C (UV-C, 30 J/m^2^) and allowed to incorporate 5-ethynyl-2’-deoxyuridine (EdU), a thymidine analog, into newly synthesized DNA for 1.5 h during DNA repair synthesis (Fig. 1A and Supplementary Fig. 1B). After click-labeling of EdU-containing DNA patches with biotin, proteins adjacent to the nascent DNA were pulled down by streptavidin affinity purification. Immunoblot analysis of pulled-down proteins showed successful capture of factors involved in DNA repair synthesis, including proliferating cell nuclear antigen (PCNA) and replication factor C (RFC) subunit 4 (RFC4), only after UV-C irradiation (Supplementary Fig. 1C).

Using mass spectrometry analysis of the proteins pulled down on a large scale, we identified proteins spatially associated with DNA repair synthesis during the repair of UV-induce DNA lesions (Fig. 1B). Several of the identified proteins have been shown to have roles in DNA synthesis (RFC1, 3, 4, PCNA), BER (APE1), DNA organization (HMGA1, HMGB1), and chromatin remodeling (CHD4, BAZ1B, SMARCA5). The chromatin remodelers CHD4, BAZ1B and SMARCA5 have been shown to function in NER [17–19]. Our screening did not detect the components of the NER pre-incision complex, possibly due to a decreased association or active dissociation of the components of the NER preincision complex during DNA repair synthesis. Among the identified proteins, we focused on DEK and NUMA1, because both proteins are implicated in DNA repair [14–16, 20], but a role in NER has not previously been defined. In addition, DEK and NUMA1 were not identified as proteins associated with nascent DNA in previous studies that employed EdU or biotin-dUTP labeling [21–23]. Consequently, it appears that DEK and NUMA1 were not detected due to their general presence at sites of unscheduled DNA synthesis (UDS) or their affinity for EdU.

We first confirmed the spatial association of DEK and NUMA1 with UV-induced DNA repair synthesis using proximity ligation assays (PLA) between each protein and click-biotinylated EdU upon UV-C irradiation [24] (Fig. 1C–F). Similar to PCNA, which served as a positive control (Fig. 1D), the number of PLA foci between DEK or NUMA1 and click-biotinylated EdU was increased upon UV-C irradiation in G1-arrested U2OS cells (Fig. 1E, F). In addition, immunofluorescence analysis revealed an increased association of DEK and NUMA1 on UV-C irradiated chromatin in G1-arrested U2OS cells (Supplementary Fig. 1D–G). To determine if DEK and NUMA1 are involved in the repair of UV-induced DNA lesions, we conducted clonogenic survival assay in multiple cancer cell lines. Depletion of DEK or NUMA1 by small interfering RNA (siRNA) increased UV sensitivity in U2OS cells (Fig. 1G, H), and a similar but moderate UV sensitivity was observed in DEK- or NUMA1-depleted XPA mutant patient cells (XP2OS) complemented with wild-type XPA (XP2OS ( + XPA) cells) (Supplementary Fig. 2A, B). Interestingly, co-depletion of DEK and NUMA1 showed comparable UV sensitivity to single depletion of each protein (Fig. 1I, J), suggesting a cooperative function of DEK and NUMA1 in repair of UV-induced DNA lesions. The sensitivity was less pronounced than that of XPA cells, in line with the level of sensitivity observed for a number of other chromatin remodelers [25–29], which was lower than that of core NER proteins. The cell cycle stage of the cells has been reported to affect UV sensitivity, with a maximum sensitivity during the S phase [30]. The cell cycle profiles of both U2OS and XP2OS ( + XPA) cells were differentially affected by DEK or NUMA1 depletion, with the most pronounced effect in U2OS cells after DEK depletion (Supplementary Fig. 2C, D). However, because all effects are measured as a reduced percentage of cells in S phase (Supplementary Fig. 2C, D), we speculate that these effects may not be related to an increase in UV sensitivity in DEK- or NUMA1-depleted cells. Taken together, we found that DEK and NUMA1 contribute to the repair of UV damage repair in a cooperative manner, and loss of DEK or NUMA1 results in increased sensitivity to UV exposure.

### DEK or NUMA1 depletion delays repair of UV-induced DNA lesions

**Fig. 2:**
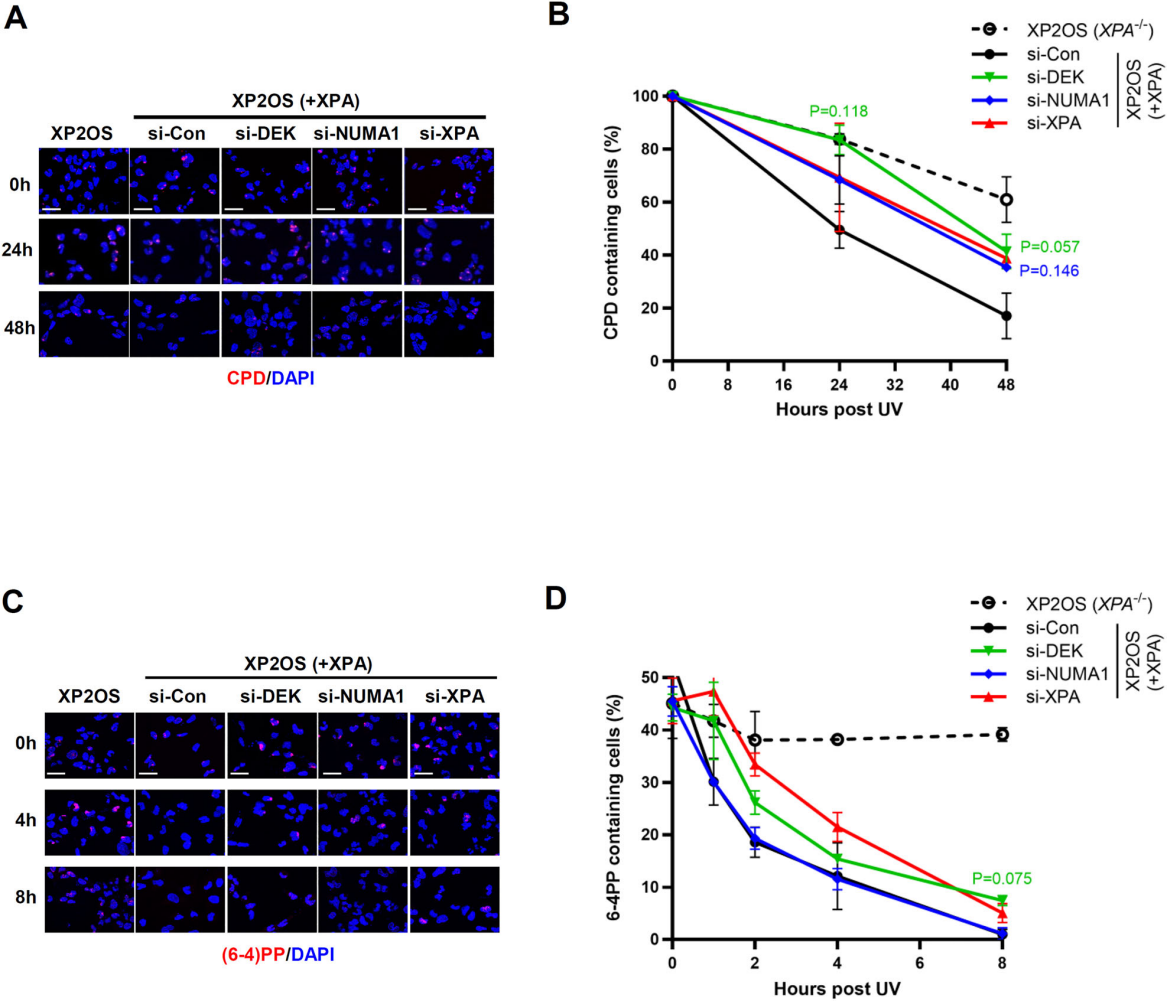
DEK- or NUMA1-depleted cells showed defects in repair of UV lesions. **A–D** XP2OS and XP2OS ( + XPA) cells were transfected with *DEK, NUMA1* or *XPA* siRNAs. 48 h after transfection, cells were irradiated through a 5 µm micropore filter with 100 J/m^2^ of UV-C, recovered for the indicated time points, and fixed for CPD immunostaining **A**, **B** or (6-4)PPs immunostaining **C**, **D**. **A**, **C** Representative images of repair kinetics for CPDs **A** and (6-4)PPs **C**. Scale bars, 50 μm. **B**, **D** Quantification of CPDs positive nuclei (%) **B** and (6-4)PPs positive nuclei (%) **D**. More than 100 cells were quantified for each repeat, each colored asterisk represents the statistical analysis in comparison to si-Con. Error bars represent SEM (*n* = 2). Statistical analysis: one-way ANOVA **B**, **D**.

UV-C irradiation is the primary source of CPDs and (6-4)PPs, which are mainly repaired by NER [1, 31]. We investigated whether the depletion of DEK or NUMA1 affects the repair of these two types of UV-induced DNA lesions. We locally irradiated with 100 J/m^2^ UV-C through membrane filter with 5 µM pores, and found that as expected, CPDs and (6-4)PPs were repaired in XP2OS ( + XPA) cells at 48 h and 8 h, respectively, while they were not repaired in XP2OS cells in that time frame [32] (Fig. 2A–D). There was an about two-fold reduction of CPDs removal in cells depleted for DEK or NUMA1 and an 1.5-fold decrease in removal of (6-4)PPs by DEK depletion (Fig. 2A–D). XP2OS ( + XPA) cells depleted for XPA by siRNA showed less effect in terms of lesion repair compared to XP2OS cells, which may be due to the residual XPA functionality in cells overexpressing XPA, as reported [33]. Interestingly, the repair defect was less pronounced when assessed by slot blot assay in cells globally irradiated with 5 J/m^2^ UV-C irradiation. Under these conditions, we only observed delayed repair of CPDs in the NUMA1-depleted cells, and the repair of (6-4)PPs was not significantly affected (Supplementary Fig. 3A–D). Considering a report showing an equivalent total number of CPDs and (6-4)PPs produced by UV-C irradiation at either 100 J/m^2^ through a filter or at 5 J/m^2^ globally [34], we propose that the differences in the density of DNA lesion may explain the divergent results in DEK- or NUMA1-depleted cells. Taken together, our results suggest that that DEK and NUMA1 play role in removal of (6-4)PPs and CPDs.

### DEK or NUMA1 depletion delays UV-induced DNA synthesis

**Fig. 3:**
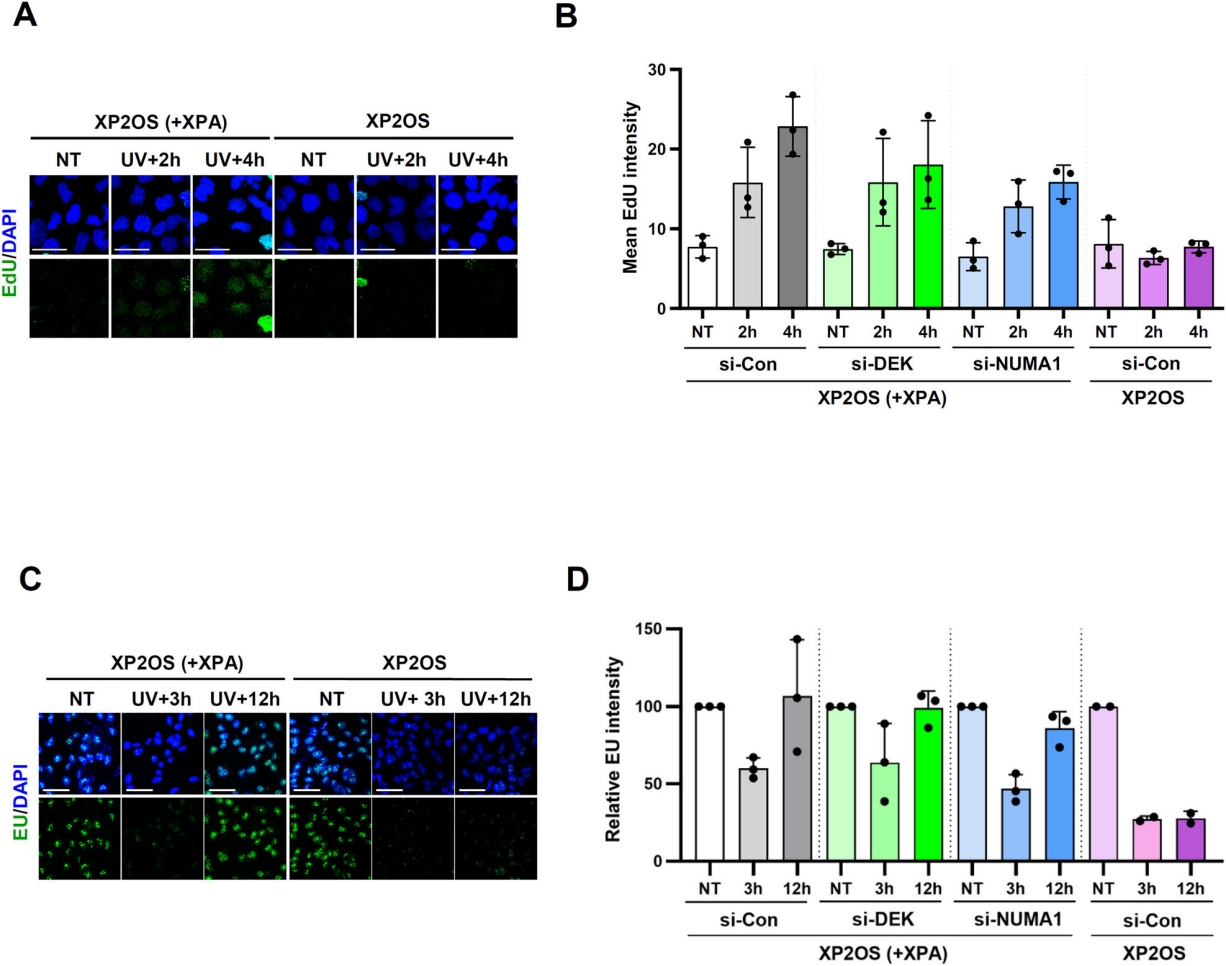
DEK- or NUMA1-depleted cells showed a slight delay in UV-induced DNA synthesis. **A, B** XP2OS and XP2OS ( + XPA) were transfected with *DEK* or *NUMA1* siRNAs. 48 h after transfection, cells were treated with CDC7i for 45 min, irradiated with 20 J/m^2^ of UV-C and recovered for the indicated time with EdU incorporation. The non-treated (NT) cells were incubated with EdU for 4 h. The cells were then subjected to a UDS assay. **A** Representative images. Scale bar, 50 μm. **B** Quantification of mean EdU intensity. Cells in S phase were identified by strong EdU signals and excluded from the analysis. Error bars represent SEM (*n* = 3). **C**, **D** XP2OS and XP2OS ( + XPA) cells were transfected with *DEK* or *NUMA1* siRNAs. 48 h after transfection, cells were irradiated with 15 J/m^2^ of UV-C and recovered for the indicated time. After incorporation of 100 µM EU for 1 h. The cells were then subjected to a RRS assay. **C** Representative images. Scale bar, 50 μm. **D** Quantification of EU intensity. Error bars represent SEM (*n* = 3, except XP2OS (*n* = 2)).

Both GG-NER and TC-NER are completed by UDS, where the short stretch of single-stranded DNA created by the excision of lesion-containing oligonucleotides is filled in by DNA synthesis outside of replication. Based on the reduced UV lesion repair upon DEK or NUMA1 depletion, we expected a decrease in UDS and tested this by measuring nuclear EdU incorporation after UV-C irradiation [35] (Fig. 3A). Cells in S phase were identified by strong EdU signals and excluded from the analysis (Fig. 3A). In non-S phase cells, EdU intensity was gradually increased in XP2OS ( + XPA) cells at the 2 h and 4 h post UV, while no signal increase was found in XP2OS cells (Fig. 3A, B). When DEK or NUMA1 was depleted in XP2OS ( + XPA) cells, the increase in EdU intensity was also observed, but showing a moderate reduction in net intensity compared to control cells at the 4 h time point, albeit without reaching statistical significance (Fig. 3A, B).

UDS is primarily a measure of GG-NER as the contribution of TC-NER to total NER activity is low [35]. Instead TC-NER can be assessed by recovery of transcription following UV irradiation [36]. As expected, RNA synthesis, as measured incorporation of 5-ethynyl uridine (EU) into nascent RNA, was decreased at 3 h, and fully restored 12 h post UV irradiation in the TC-NER proficient XP2OS ( + XPA) cells, while no recovery of RNA synthesis was observed in XP2OS cells at the same time point (Fig. 3C, D). In DEK- or NUMA1-depleted cells, like control cells, RNA synthesis was fully restored 12 h after UV irradiation (Fig. 3C, D). Taken together, these results suggest that there is a delay in the completion of GG-NER, while TC-NER is not significantly affected in DEK- or NUMA1-depleted cells.

Both DEK and NUMA1 have been reported to regulate gene expression [12, 37] and NUMA1 is known to interact with chromatin remodelers and transcription factors [16, 38]. Therefore, it is possible that altered expression of NER genes due to depletion of either DEK or NUMA1 could be responsible for the observed repair defects. However, we did not observe a change in the levels of the core NER factors by DEK or NUMA1 depletion in U2OS or XP2OS ( + XPA) cells (Supplementary Fig. 4A–D). Although we cannot exclude that the transcriptional regulation of other NER-associated genes is affected, these results support the conclusion that DEK and NUMA1 directly affect UV lesion repair.

### DEK or NUMA1 depletion delays XPF recruitment to UV-induced DNA damage

**Fig. 4:**
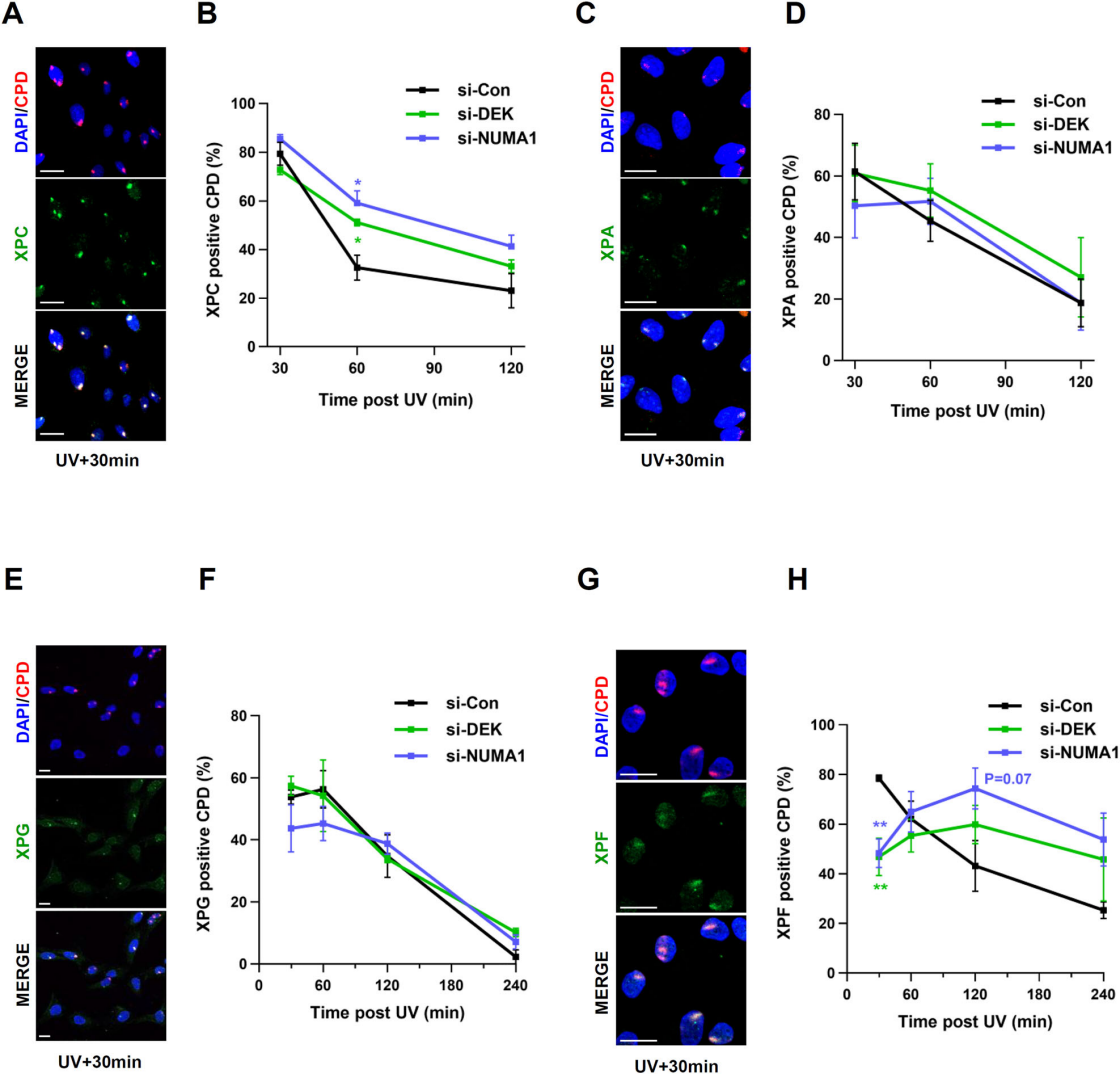
DEK- or NUMA1-depleted cells showed abnormal XPF recruitment kinetics. **A–D** U2OS cells were transfected with *DEK* or *NUMA1* siRNAs. 48 h after transfection, cells were irradiated through a 5 µm micropore filter with 100 J/m2 of UV-C and fixed at the indicated time points. The cells were then immunostained for CPD and XPC **A**, **B**, XPA **C**, **D**, XPG **E**, **F**, or XPF **G**, **H**. **A**, **C**, **E**, **G** Representative images. Scale bar, 20 μm. **B**, **D**, **F**, **H** Quantification of co-localization with CPDs (%). Each colored asterisk represents the statistical analysis in comparison to si-Con. Error bars represent SEM (*n* > 2). Statistical analysis: one-way ANOVA, **P < 0.01, *P < 0.05, and otherwise not significant.

We next sought to delineate the step and molecular mechanism of how DEK and NUMA1 contribute to NER. We first asked whether DEK and NUMA1 influence the dynamics of the core NER factors around UV-induced DNA lesions. We measured the percentage of co-localization of XPC, XPA, XPG and XPF with locally induced UV DNA lesions in wild-type and DEK- and NUMA1-depleted cells. In U2OS cells, the XP proteins co-localized with CPDs, which we use as a marker for sites of UV-induced DNA lesions, within 30 min, and the percentage of co-localization gradually decreased at later time points (Fig. 4A–H). When DEK or NUMA1 was depleted, the co-localization of the damage sensor XPC with CPDs was not altered at 30 min repair time. However, the co-localization remained higher at 1 and 2 h repair time in DEK- or NUMA1-depleted cells (Fig. 4A, B). Recruitment and release of XPA and XPG, components of the NER preincision complex, were not affected by depletion of DEK and NUMA1 (Fig. 4C–F). Interestingly, the percentage of co-localization of XPF to CPDs showed the most striking differences (Fig. 4G, H). In DEK- or NUMA1-depleted cells, the co-localization of XPF to CPDs was lower than control cells at 30 min, gradually increased up to 2 h, and then decreased at 4 h (Fig. 4G, H). These observations suggest that the association of XPF with UV lesion and its subsequent dissociation are delayed in DEK- or NUMA1-depleted cells.

### DEK or NUMA1 depletion leads to RFC-dependent PCNA enrichment in UV lesions

**Fig. 5:**
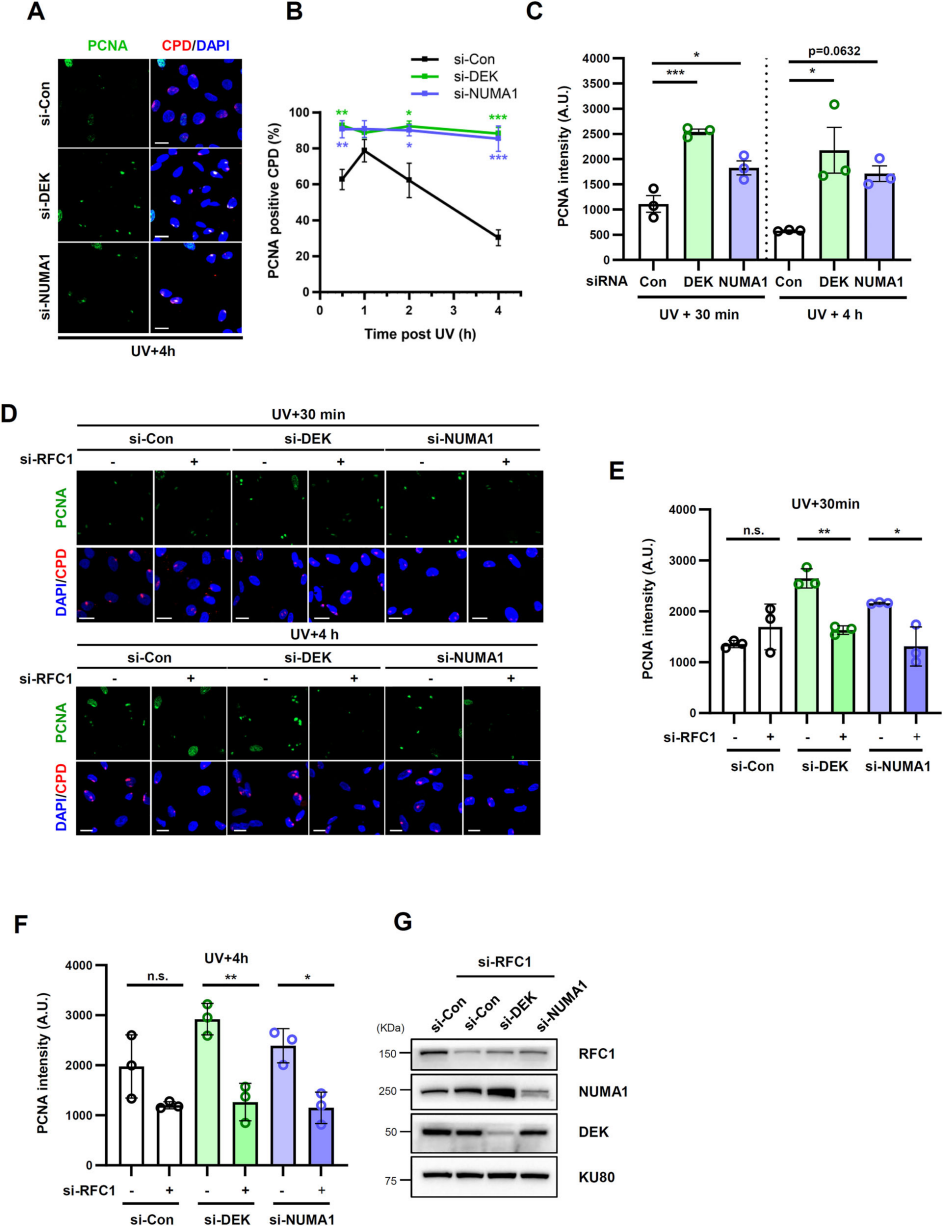
DEK- or NUMA1-depleted cells showed abnormal PCNA accumulation. **A–C** U2OS cells were transfected with *DEK* or *NUMA1* siRNAs. 48 h after transfection, cells were irradiated through a 5 µm micropore filter with 100 J/m^2^ of UV-C, fixed at the indicated time points, and immunostained for PCNA and CPD. **A** Representative images of PCNA co-localization with CPDs. Scale bar, 20 μm. **B** Quantification of PCNA co-localization with CPDs (%). Each colored asterisk represents the statistical analysis in comparison to si-Con. Quantification of PCNA signal intensity at CPD foci. A.U., arbitrary unit. **B**, **C** S phase cells with S phase-specific PCNA foci staining pattern were excluded from the analysis. Error bars represent SEM (*n* = 3). **D–G** U2OS cells were transfected with siRNAs as indicated. 48 h after transfection, cells were irradiated through a 5 µm micropore filter with 100 J/m^2^ of UV-C and subjected to immunostaining **D–F** or lysed for Western blot **G**. **D** Representative images. Scale bar, 20 μm. **E**, **F** Quantification of PCNA signal intensity at CPD foci (A. U.). S phase cells with S phase-specific PCNA foci staining pattern were excluded from the analysis. Images from the 30 min **E** and 4 h **F** time points were analyzed using the same parameters, and the graphs are shown separately for each time point. Error bars represent SEM (*n* = 3). Statistical analysis: one-way ANOVA **B**, **C**, **E**, **F**, ***P < 0.005, **P < 0.01, *P < 0.05, and ns not significant.

After the first 5´ cut by XPF, nascent DNA synthesis fills the incision gap [4–6]. Based on delayed UDS and XPF recruitment to UV lesions in DEK- or NUMA1-depleted cells, we asked whether DEK and NUMA1 affect the dynamics of PCNA, a DNA polymerase processivity factor, to the UV-induced DNA lesions in non-S phase cells (Fig. 5A). In control cells, PCNA co-localization with CPDs reached a peak at 1 h repair time and gradually decreased until 4 h (Fig. 5B). Unexpectedly, the co-localization of PCNA with CPDs was higher in DEK- or NUMA1-depleted cells compared to control cells at all time points from 30 min to 4 h (Fig. 5A, B). Quantitative analysis of the signal intensity of PCNA co-localized with CPD clearly showed an increase after DEK or NUMA1 depletion at both 30 min and 4 h time points (Fig. 5C).

PCNA homotrimers are primarily loaded onto DNA by the RFC complex [39, 40]. To determine if PCNA accumulation at UV-induced DNA lesions after DEK or NUMA1 depletion depended on RFC-mediated loading, we examined the effect of RFC1 depletion (Fig. 5D–G). In control cells, RFC1 depletion did not significantly alter PCNA signal intensity at UV lesions at either early (30 min) or late (4 h) time points, suggesting that a substantial amount of PCNA is associated with UV lesions independently of RFC, consistent with previous findings [41]. However, the increased PCNA accumulation at UV lesions observed in DEK- or NUMA1-depleted cells was reduced to control levels when RFC1 was co-depleted at both time points (Fig. 5D–F). This indicates that the accumulated PCNA observed in DEK- or NUMA1-depleted cells is loaded in an RFC-dependent manner, in contrast to the apparent RFC-independent association of UV lesions in control cells.

### Early and late PCNA accumulation after DEK or NUMA1 depletion are the result of distinct repair pathways

The DNA substrate for PCNA loading, a double-stranded DNA/single-stranded DNA junction with a free 3’-hydroxyl (OH) group, is generated by the 5’ incision by XPF in NER [4]. To investigate the dependency of PCNA localization at UV lesions on the NER pathway, we used *XPF* knockout (KO) cells (Supplementary Fig. 5A–D). We first confirmed that a comparable number of CPDs were formed at different time points in wild-type and *XPF* KO cells (Supplementary Fig. 5B, C). PCNA did not co-localize of with CPDs 30 min after UV irradiation in *XPF* KO U2OS cells (Supplementary Fig. 5B, D), consistent with the previously reported requirement of the XPF-mediated 5’ incision for PCNA localization to CPDs [4]. Interestingly, co-localization of PCNA with UV lesions at the 4 h time point in *XPF* KO cells was comparable to that of wild-type cells (Supplementary Fig. 5B, D), suggesting that the localization of PCNA at later time points occurs independent of NER.

**Fig. 6:**
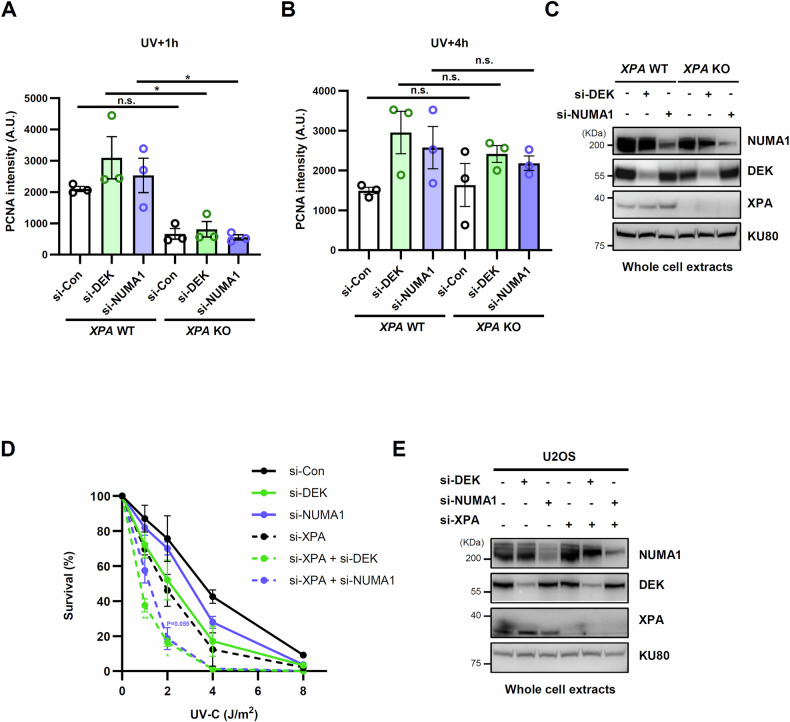
Early and late PCNA accumulation after DEK or NUMA1 depletion showed differential dependence on XPA. **A–C** *XPF* WT and KO U2OS cells were transfected with *DEK* or *NUMA1* siRNAs. 48 h after transfection, cells were irradiated through a 5 µm micropore filter with 100 J/m^2^ of UV-C, recovered, and subjected to immunostaining for CPD and PCNA **A**, **B** or lysed for Western blot **C**. **A**, **B** Quantification of PCNA signal intensity at CPD foci (A. U.). S phase cells with S phase-specific PCNA foci staining pattern were excluded from the analysis. Images from the 30 min and 4 h time points were analyzed using the same parameters, and the graphs are shown separately for each time point. Error bars represent SEM (*n* = 3). **D**, **E** U2OS cells were transfected with *DEK, NUMA1*, and/or *XPA* siRNAs as indicated. 48 h after transfection, cells were irradiated with UV-C and subjected to clonogenic survival assay **D** or lysed for Western blot **E**. **D** Each colored asterisk represents statistical analysis in comparison to si-XPA. Error bars represent SEM (*n* = 3). Statistical analysis: one-way ANOVA **A**, **B**, **D**, **P < 0.01, *P < 0.05, and ns, not significant.

We next asked whether DEK or NUMA1 depletion affected the NER-independent accumulation of PCNA at UV lesions observed (Fig. 6A–C). For this purpose, we used *XPA* KO cells, in which we confirmed NER-independent localization of PCNA to UV lesions at 4 h post UV (Fig. 6A, B and Supplementary Fig. 6). PCNA marginally co-localized with UV-induced DNA lesions at 1 h post UV in *XPA* KO cells, whereas the co-localization was comparable in wild-type and *XPA* KO cells at 4 h post UV. This was unexpected as there was no increase in EdU at 4 h post UV in *XPA* KO cells (Fig. 3B). The relatively small patch size associated with the non-NER pathway may limit the incorporation and subsequent detection of EdU during DNA repair synthesis. Interestingly, we found that DEK- or NUMA1-depletion increased PCNA accumulation at UV lesions in *XPA* KO cells only at the 4 h but not 1 h post UV (Fig. 6A, B). This suggests that in DEK- or NUMA1-depleted cells, early, but not late PCNA accumulation at UV lesions is associated with NER.

Excessive PCNA accumulated on DNA has been reported to interfere with various aspects of DNA metabolism [42, 43]. Similarly, PCNA accumulation at UV lesions following DEK or NUMA1 depletion may cause the delay in the repair of UV lesions and UV-induced DNA repair synthesis. Our results suggest that non-NER repair pathways contribute to the late accumulation of PCNA in the absence of DEK or NUMA1 and that these pathways may contribute to UV resistance independently of NER. To test this possibility, we examined UV-C sensitivity by co-depleting XPA with DEK or NUMA1. To avoid masking the effects of co-depleting DEK or NUMA1, we first titrated the XPA siRNA concentration. As a result, we found that co-depletion indeed resulted in additive UV-C sensitivity (Fig. 6D, E). Taken together, these results suggest a defect in a late, non-NER repair pathways are responsible for the increased UV sensitivity in DEK- or NUMA1-depleted cells.

### DEK or NUMA1 shows an epistatic relationship with APE1 for UV sensitivity

**Fig. 7:**
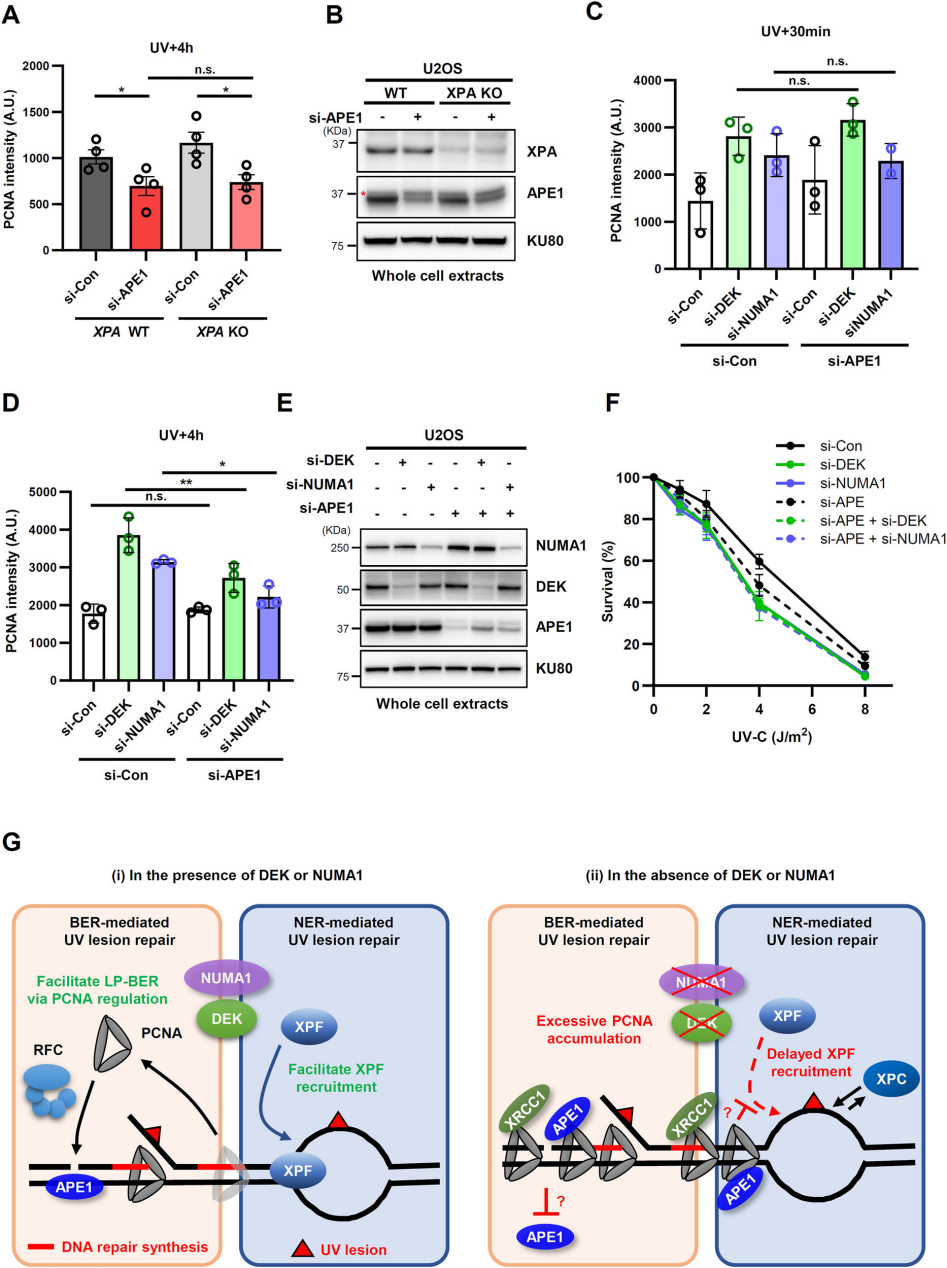
Late PCNA accumulation after DEK or NUMA1 depletion was dependent on APE1. **A–E** *XPA* WT and KO U2OS cells **A**, **B** and U2OS cells **C–E** were transfected with siRNAs as indicated. 48 h after transfection, cells were irradiated through a 5 µm micropore filter with 100 J/m^2^ of UV-C, recovered for the indicated times, and subjected to immunostaining for CPD and PCNA **A**, **C**, **D** or lysed for Western blot **B**, **E**. **B** Red asterisk indicates non-specific band. **A**, **C**, **D** Quantification of PCNA signal intensity at CPD foci (A. U.). S phase cells with S phase-specific PCNA foci staining pattern were excluded from the analysis. **C**, **D** Images from the 30 min and 4 h time points were analyzed using the same parameters, and the graphs are shown separately for each time point. Error bars represent SEM (*n* = 4 **A**, *n* = 2 **C**, **D**). **F** U2OS cells were transfected with *DEK, NUMA1*, and/or *APE1* siRNAs as indicated. 48 h after transfection, cells were irradiated with UV-C and subjected to clonogenic survival assay. Error bars represent SEM (*n* = 5). **G** Graphic model illustrating the role of DEK and NUMA1 in repairing UV-induced DNA lesions. Statistical analysis: one-way ANOVA **A**, **C**, **D**, ****P < 0.001 ***P < 0.005, **P < 0.01, *P < 0.05, and ns not significant.

Our mass spectrometry analysis identified APE1, a core BER protein, to be associated with UV-induced DNA repair synthesis (Fig. 1B), raising the possibility of an involvement of this BER endonuclease in the repair of UV-induced DNA damage. This observation is consistent with a recent report showing that APE1-mediated LP-BER can remove residual UV lesions in NER-deficient cell lines [11]. Therefore, we tested whether BER is related to UV lesion repair in DEK- or NUMA1-depleted cells. APE1 depletion reduced the amount of PCNA co-localization with CPDs at 4 h post UV in the absence of XPA (Fig. 7A, B), indicating that PCNA accumulation at UV lesions is partially dependent on APE1-mediated LP-BER at the late stage. In addition, the increased PCNA accumulation at UV lesions in the absence of DEK or NUMA1 was partially but significantly reduced by APE1 co-depletion at the 4 h but not 0.5 h post UV (Fig. 7C–E). This suggests that the accumulation of PCNA in the absence of DEK or NUMA1 is mediated by APE1-mediated LP-BER.

Based on the APE1-dependent PCNA accumulation and the contribution of non-NER pathways to UV repair involving DEK or NUMA1 depletion (Fig. 6D), we tested whether APE1- depletion would lead to additional sensitivity of DEK- or NUMA1-depleted cells to UV-C irradiation. We found that co-depletion of APE1 with DEK or NUMA1 did not result in additional UV-C sensitivity compared to APE1 depletion alone (Fig. 7F). This indicates that DEK or NUMA1 share an epistatic relationship with APE1-mediated LP-BER, rather than with NER, in terms of UV sensitivity.

## Discussion

In this study, we used a click chemistry-based proteomic approach in G1 to identify proteins associated with the repair synthesis of gaps generated during the repair of UVC-induced DNA lesions. We identified numerous proteins previously known to be involved in NER or BER validating our approach. Among the identified proteins that have not yet been shown to be involved in the repair of UV lesions were DEK and NUMA1. These two proteins have been reported to be involved in the regulation of DNA double-strand break repair or oxidative damage repair [14–16, 20]. Following up on our observation that DEK and NUMA1 are associated with sites of UV-induced DNA repair synthesis, we uncovered that they contribute to the cellular UV resistance by promoting both NER and APE1-mediated BER.

In the context of NER, DEK and NUMA1 appear to affect the association of the XPC and XPF proteins with repair complexes (Fig. 4). The damage sensor XPC has been reported to dissociate from DNA lesions concomitantly with XPG recruitment, and a delay in XPC release may thus result in reduced XPG recruitment [44, 45]. However, the XPG kinetics are unaltered in DEK- or NUMA1 -depleted cells (Fig. 4F), suggesting that XPC dissociation is intact and thus not likely to be the reason for the increased co-localization of XPC with CPDs (Fig. 4B). XPF recruitment requires the presence of XPA, RPA and XPG as well as the helicase activity of TFIIH [32, 46–49]. Considering that there is no change in XPA and XPG localization at CPDs upon DEK or NUMA1 depletion (Fig. 4D, F), DEK or NUMA1 may be involved in facilitating XPF binding to UV lesions (Fig. 7G, I). The persistent co-localization of XPCs with CPDs after DEK or NUMA1 depletion (Fig. 4B) therefore likely reflects the repeated binding and dissociation of XPC from lesions that remain due to delayed XPF recruitment [50]. It is known that XPC dissociates from DNA lesions upon assembly of the full preincision complex and thus departs earlier from UV lesion than the remaining NER complex and the persistence of XPC lesion likely reflects this property (Fig. 7G, ii).

Since XPF incision is the trigger for repair synthesis, the delayed XPF activity observed in DEK- or NUMA1-depleted cells may affect NER repair synthesis and thus explain the identification of DEK and NUMA1 as proteins associated with repair synthesis. Interestingly, however, co-depletion of XPA with DEK or NUMA1 resulted in additive UV sensitivity (Fig. 6D), suggesting that DEK and NUMA1 contribute to UV resistance outside of NER as well. In light of a recent report that LP-BER can serve as an alternative mechanism for repairing UV-induced DNA lesions [11] and our finding that APE1 is associated with UV-induced DNA repair synthesis (Fig. 1B), we explored a possible role of DEK and NUMA1 in LP-BER of UV lesions (Fig. 1B). Indeed, co-depletion of APE1 with DEK or NUMA1 did not result in further UV sensitivity (Fig. 7F), indicating that defective LP-BER, rather than NER, is responsible for the increased UV sensitivity in DEK- or NUMA1-depleted cells.

We sought to get insight into the mechanism by which the LP-BER is impaired after DEK or NUMA1 depletion. We observed that PCNA accumulation at UV lesions at late repair time (4 h) is dependent on APE1, but not on XPA in DEK- or NUMA1-depleted cells (Figs. 6A, B, 7C, D). In BER, APE1 catalyzes the cleavage of the phosphodiester bond on the 5′ side an abasic site, creating a break with 3′-OH at the end, a DNA substrate for PCNA loading [51, 52]. Considering the PCNA accumulation after DEK or NUMA1 depletion (Fig. 5H), APE1 may generate more nicks for PCNA loading under these conditions. Consistent with the epistatic relationship of APE1 and DEK/NUMA1, the increased PCNA accumulation under these conditions may be harmful. It is known that PCNA over accumulation on DNA can disrupt various DNA metabolic processes by spatially occupying the region of DNA process taking place or keeping capture of proteins associated with the DNA process required [42, 43]. In a biochemically reconstituted system, the stimulation of LP-BER by APE1 was reported to be inhibited by the presence of PCNA [53]. We propose that excessive accumulation of PCNA on DNA at nicks generated by APE1 in the absence of DEK or NUMA1 depletion may hinder the action of other APE1 proteins initiating BER in the surrounding area (Fig. 7G, ii). In addition, since APE1, as well as other key BER proteins such as XRCC1, physically interacts with PCNA [54, 55], their excessive binding to the accumulated PCNA may negatively affect the BER process (Fig. 7G, ii). Further studies are needed to determine whether and how DEK or NUMA1 regulate the loading or unloading of PCNA at UV lesions and how PCNA accumulation affects the LB-BER process.

The early accumulation of PCNA at CPD sites following UV irradiation is dependent on NER activity (Fig. 6A). Consistent with this, XPF was detected at CPD sites as early as 30 min post-UV exposure (Fig. 4H). Interestingly, in DEK- or NUMA1-depleted cells, a significant proportion of PCNA signals appeared at CPD sites within 30 min, despite the absence of detectable XPF recruitment (Fig. 4H and Fig. 5B). The origin of these early PCNA signals therefore remains uncertain. One possible explanation is that they may represent pre-loaded PCNA molecules that persist at chromatin after UV treatment [6], or that PCNA is loaded independently of RFC activity [41]. However, the present study did not directly address these alternative mechanisms, which require further investigation.

Our results highlight the importance of DEK and NUMA1 in maintaining genomic stability by influencing NER- and LP-BER of UV-induced DNA lesions. The epistatic relationship with APE1 suggests that DEK and NUMA1 may serve as regulators in coordinating repair pathways to ensure proper initiation and progression of BER following UV damage. Given the important roles of DEK and NUMA1 in other DNA repair pathways, such as DSB repair and oxidative damage repair, it will be of interest to see if DEK and NUMA1 regulate distinct DNA repair pathways by a common mechanism possibly be regulating the status of PCNA binding in various pathways.

## Material and methods

### Cell lines

The following cell lines were utilized in this study: SV40-transformed human fibroblasts XP2OS (XPA mutant), XP2OS-2-18 (XP2OS cells complemented with wild-type XPA, referred to as XP2OS ( + XPA)), U2OS, U2OS-*XPA* KO, and U2OS-*XPF* KO cells. Cells were cultured in Dulbecco’s Modified Eagle’s Medium (DMEM) supplemented with 10% fetal bovine serum (FBS, Millipore) and penicillin-streptomycin (Gibco) in a humidified atmosphere of 5% CO_2_ at 37 °C. The cells were tested periodically for mycoplasma contamination.

### siRNA transfection

siRNA transfection was conducted using Lipofectamine^TM^ RNAiMAX transfection reagents (Thermo Fisher Scientific), in accordance with the manufacturer’s instructions. Six hours after transfection, the medium containing the transfection reagent was replaced with fresh medium. siRNA was treated with 20 nM as the final concentration, except for the XPA co-depletion clonogenic survival assay where 7.5 nM of si-XPA was used as the final concentration. The following synthetic duplex siRNAs were purchased from Bioneer: si-Control (#SN-1002), si-APE1 (5’-GUCUGGUACGACUGGAGU-3’), si-DEK (5’-CUCUGUAAAAGUGUCUGUA-3’), si-NUMA1 (5’-CUCUUGGGUGAACAGUCUA-3’); si-RFC1 (5’-GAAGGCGGCCUCUAAAUCAUU-3’), and si-XPA (5’-CAGAGAUGCUGAUGAUAAA = UU-3’). ON-TARGETplus SMARTpool si-APE1 (#L-010237-00-0005) was purchased from Dharmacon.

### Antibodies

The following antibodies were utilized in this study: anti-α-TUBULIN (T9026, Sigma-Aldrich); anti-DEK (ab166624, Abcam); anti-NUMA1 (ab97585, Abcam; sc-365532, Santa Cruz); anti-CPD (CAC-NM-DND-002, Cosmobio); anti-(6-4)PPs (CAC-NM-DND-001, Cosmobio); anti-XPC (sc-74411, Santa Cruz; A301-121A, Bethyl Laboratories); anti-XPB (PA5-22254, Thermo Fisher Scientific); anti-XPD (ab54676, Abcam); anti-p62 (sc-48431, Santa Cruz); anti-XPA (sc-853 and sc-28353, Santa Cruz); anti-XPF (NBP2-58407, Novus Biologicals); anti-XPG (NB100-74611, Novus Biologicals; A301-484A, Bethyl Laboratories); anti-PCNA (ab18197, Abcam); anti-RFC1 (NBP2-54960, Novus Biologicals); anti-APE1 (ab189474, Abcam); anti-KU80 (MA512933, Invitrogen); anti-Biotin (sc-57636, Santa Cruz); anti-Histone H3 (07-690, Merck Millipore); Goat anti-Rabbit IgG (H + L) Cross-Adsorbed Secondary Antibody, Alexa Fluor^TM^ 488 (A11008, Thermo Fisher Scientific); Cy3-Goat Anti-Mouse IgG (115-65-146, Jackson Immunoresearch); Horseradish peroxidase (HRP)-conjugated Goat anti-rabbit IgG antibody (ADI-SAB-300-J, Enzo); HRP-conjugated Goat anti-mouse IgG antibody (ADI-SAB-100-J, Enzo).

### Isolation of proteins associated with UV-induced DNA synthesis

U2OS cells were seeded on 150 mm culture dishes. The next day, cells were treated with 1 µM CDK4/6i (PD0332991, Selleckchem) for 24 h to arrest cell cycle in the G1 phase and treated with 1 µM CDC7i (PHA767491, Selleckchem) for 45 min to block new origin firing. Cells were then irradiated with 30 J/m^2^ of UV-C, and 20 µM 5-ethynyl-2′-deoxyuridine (EdU) with 1 µM 5’-deoxy-5-fluorouridine was incorporated for 1.5 h to label UV-induced nascent DNA synthesis. Cells were then fixed with 1% formaldehyde for 20 min and stored at -80 °C. Subsequent cell lysis, EdU click reaction with biotin-azide, and streptavidin-agarose pull-down were performed as described in [56], which was used for the iPOND assay.

### Liquid chromatography-mass spectrometry (LC-MS)

The eluted protein was subjected to sodium dodecyl sulfate (SDS)-polyacrylamide gel electrophoresis (PAGE) and the gel was stained with Imperial Protein stain (Thermo Fisher Scientific) for 1 h. After subsequent destaining, protein bands were excised, excised protein bands were cut into small sections and subjected to in-gel digestion with trypsin. The tryptic digests were separated by online reversed-phase chromatography using a Eazy nano LC 1200 UHPLC (Thermo Fisher Scientific) equipped with an autosampler using a reversed-phase peptide trap Acclaim PepMap™ 100 (75 μm inner diameter, 2 cm length, Thermo Fisher Scientific) and a reversed-phase analytical column PepMap™ RSLC C18 (75 μm inner diameter, 15 cm length, 3 μm particle size, Thermo Fisher Scientific), followed by electrospray ionization at a flow rate of 300 nl/min. The chromatography system was coupled in line with an Orbitrap Fusion Lumos mass spectrometer (Thermo Fisher Scientific). All MS/MS samples were analyzed using Sequest (XCorr Only) (Thermo Fisher Scientific; version IseNode in Proteome Discoverer 2.2.0.388) and X! Tandem (The GPM, thegpm.org; version CYCLONE (2010.12.01.1)). Sequest (XCorr Only) was set up to search uniprot-human-2017-01_DB2_contam.fasta (unknown version, 42230 entries) assuming the digestion enzyme trypsin. X! Tandem was set up to search a reverse concatenated subset of the uniprot-human-2017-01_DB2_contam database (unknown version, 34216 entries) (only “Mudpit_Untreated1: Untreated1”) assuming trypsin and a reverse concatenated subset of the uniprot-human-2017-01_DB2_contam database (unknown version, 36128 entries) (only “Mudpit_Treated2: Treated1”) assuming trypsin. Sequest (XCorr Only) and X! Tandem were searched with a fragment ion mass tolerance of 0.80 Da and a parent ion tolerance of 10.0 PPM.

### Criteria for the identification of peptides and proteins

Scaffold (version Scaffold_4.11.0, Proteome Software Inc.) was used to validate MS/MS-based peptide and protein identifications. Peptide identifications were accepted if they could be established at greater than 95.0% probability. Peptide probabilities from X! Tandem were assigned by the Scaffold Local FDR algorithm. Peptide probabilities from Sequest (XCorr Only) were assigned by the Peptide Prophet algorithm [57] with Scaffold delta-mass correction. Protein identifications were accepted if they could be established at greater than 95.0% probability and contained at least 2 identified peptides. Protein probabilities were assigned by the Protein Prophet algorithm [58]. Proteins that contained similar peptides and could not be differentiated based on MS/MS analysis alone were grouped to satisfy the principles of parsimony. Proteins were annotated with GO terms from NCBI (downloaded 2019. 5. 14) [59].

### Proximal ligation assay (PLA) of biotin-clicked EdU and proteins of interest

PLA-based detection of proteins associated with UV-induced DNA synthesis was performed based on the quantitative in situ analysis to monitor the protein interactions at DNA replication forks (SIRF) assay [24] with slight modifications. In brief, cells were cultured on LabTek™ chamber slides (Thermo Fisher Scientific), arrested in G1 phase by CDK4/6i treatment, and incubated with 100 μM EdU for 1.5 h after UV-C irradiation. Cells were then fixed with 4% paraformaldehyde (PFA) in phosphate-buffered saline (PBS, pH 7.4) for 20 min and permeabilized with 0.5% Triton X-100 in PBS for 30 min at room temperature. After PBS washing, the click reaction cocktail (2 mM CuSO_4_, 20 μM biotin-azide and 10 mM sodium ascorbate in PBS) was added to each chamber and cells were incubated for 30 min at room temperature. Cells were then blocked in 10% FBS in PBS for 1 h at room temperature, and incubated with either mouse anti-biotin or rabbit anti-biotin antibodies in conjunction with the antibody for the protein of interest overnight at 4 °C. Cells were washed twice with PBS supplemented with 0.1% Tween-20 (PBS-T) and incubated with pre-mixed Duolink PLA plus and minus probes (Sigma Aldrich) for 1 h at 37 °C in a humidity chamber. Subsequent PLA steps were performed using the Duolink® PLA Fluorescence Kit (Sigma Aldrich) according to the manufacturer’s instructions. Finally, slides were stained with VECTASHILED Antifade Mounting Medium (H-1200-10, Vector laboratories) and imaged using a LSM880 confocal microscope (Carl Zeiss). The number of Foci was counted using ZEN software (Carl Zeiss).

### Immunofluorescence

Cells were fixed and stained as previously described with slight modifications [60]. To detect chromatin-associated proteins, cells were pre-extracted with cytoskeletal (CSK) buffer (10 mM PIPES, 100 mM NaCl, 300 mM sucrose, 3 mM MgCl_2_, 1 mM EGTA and 0.5% Triton X-100™) for 10 min on ice and fixed with 4% PFA for 20 min at room temperature. After washing with PBS and incubation with blocking buffer (10% FBS in PBS) for 1 h, cells were incubated with the indicated primary antibodies diluted in blocking buffer for 1 h at room temperature or overnight at 4 °C. After washing with 0.1% Triton X-100 in PBS, cells were incubated with Alexa Fluor-conjugated secondary antibodies for 1 h at room temperature. Following washing steps, the cells were mounted with antifade mounting medium with DAPI. The samples were then analyzed using an LSM880 confocal microscope (Carl Zeiss), and the mean intensity of the signal was measured using ZEN software (Carl Zeiss).

### Unscheduled DNA synthesis

To visualize unscheduled DNA synthesis, cells were treated with a CDC7i for 45 min to block new origin firing. Cells were then irradiated with 20 J/m^2^ of UV-C and allowed to recover for the indicated time points while incorporating 15 µM EdU. The unincorporated EdU was depleted by changing into fresh medium containing 10 µM thymidine for 15 min. Then the cells were fixed in 4% PFA for 20 min, permeabilized with 0.5% Triton X-100 in PBS, and subjected to a click reaction with Alexa Fluor™ 488 picolyl azide at room temperature. After thorough washing with 3% BSA supplemented PBS, cells were mounted with antifade mounting medium with DAPI for imaging. Samples were analyzed using an LSM880 confocal microscope (Carl Zeiss), and the mean intensity was measured using ZEN software (Carl Zeiss).

### Recovery of RNA synthesis (RRS)

The RRS assay was performed as previously reported [36] with slight modifications. Cells were subjected to 100 J/m^2^ UV-C irradiation and recovered for the indicated time points. RNA synthesis was marked by incorporating 100 µM 5’-ethynyl uridine (EU, ab146642, Abcam) for 1 h. Cells were then pre-extracted with CSK buffer for 5 min on ice, fixed in 4% PFA for 20 min, and subjected to a click reaction with Alexa Fluor™ 488 picolyl azide for 30 min at room temperature. After thorough washing with 3% BSA supplemented PBS, cells were mounted with antifade mounting medium with DAPI for imaging. Samples were analyzed using an LSM880 confocal microscope (Carl Zeiss), and the mean signal intensity was measured using ZEN software (Carl Zeiss).

### Local UV irradiation assay

Cells were seeded on coverslips (Neuvitro, cat# GG-22-PDL) 1 day before UV treatment. For UV treatment, cells were washed by Dulbecco’s phosphate-buffered saline (DPBS), covered with a 5 µm isopore membrane (TMTP04700, Merck), and irradiated with 100 J/m^2^ of UV-C. Following UV irradiation, cells were incubated for different repair times (0, 1, 2, 4, 8 h for (6-4)PPs repair assay and 0, 8, 24, 48 h for CPDs repair assay).

### Immunostaining of CPDs and (6-4)PPs

After local UV irradiation and recovery, cells were washed with DPBS, fixed with 4% PFA in DPBS for 10 min at room temperature, and permeabilized with DPBS containing 0.5% Triton X-100 for 5 min on ice. For the detection of CPDs and (6-4)PPs, DNA was denatured with 2 M HCl for 30 min at room temperature. Following a 30 min blocking step with DPBS containing 20% FBS, cells were incubated with diluted primary antibodies in DPBS containing 5% FBS for 2 h at room temperature. Following five washes with DPBS, the cells were incubated with diluted secondary antibody in DPBS containing 5% FBS for 1 h at room temperature and cells were mounted with antifade mounting medium with DAPI. The samples were analyzed using an inverted fluorescence microscope (Axio Observer 7, Carl Zeiss).

### Immunostaining for detecting colocalization of proteins with CPDs

After local UV irradiation and recovery, cells were lysed with cold hypotonic buffer (10 mM Tris-HCl (pH 8.0), 2.5 mM MgCl_2_, 10 mM β-glycerophosphate, 0.2% Igepal, 0.2 mM PMSF, 0.1 mM Na_3_VO_4_) for 15 min on ice and washed with hypotonic buffer without Igepal for 4 min at room temperature. The cells were fixed with 2% PFA in DPBS for 5 min at room temperature. Following two washes with 0.1% Triton X-100 in DPBS for 5 min, cells were stored in 70% ethanol at -20 °C. After removal of ethanol, the cells were pre-equilibrated with digestion buffer (10 mM Tris-HCl (pH 8.0), 5 mM MgCl_2_) for 2 min and the DNA was digested with 20U DNaseI (D4527, Sigma-Aldrich) in 1 mL of digestion buffer for 40 sec at room temperature. Subsequently, 500 mM EDTA was directly added at a final concentration of 10 mM to stop the digestion. Cells were then washed twice with 10 mM EDTA in DPBS for 5 min. After incubation with blocking buffer (1% BSA, 0.2% Tween-20), cells were incubated with diluted primary antibodies in blocking buffer for 2 h. Following three washes with DPBS containing 0.2% Tween-20, cells were incubated with diluted secondary antibody in blocking buffer for 1 h at room temperature. The cells were mounted with antifade mounting medium with DAPI for imaging. The samples were analyzed using an inverted fluorescence microscope (Axio Observer 7, Carl Zeiss) and the mean intensity of the signal was measured using ZEN software (Carl Zeiss).

### Slot blot assay

Cells were irradiated with 5 J/m^2^ of UV-C and collected after different time points (0, 2, 4, 8, 24 h for (6-4)PPs and 0, 8, 24, 48 h for CPDs). Genomic DNA was prepared from harvested cells using the QIAamp DNA mini kit (Qiagen) according to the manufacturer’s protocol. Following the adjustment of the genomic DNA concentration, the DNA was denatured with 7.8 mM EDTA (for (6-4)PPs) or with 0.4 M NaOH and 10 mM EDTA (for CPDs). The denatured DNA was boiled at 95 °C for 10 min. DNA samples (200 ng of (6-4)PPs or 100 ng of CPDs) were neutralized by adding an equal volume of 2 M ammonium acetate (pH 7.0) and vacuum-transferred to a pre-washed nitrocellulose membrane using a BioDot SF microfiltration apparatus (Bio-Rad). Each well was washed twice with SSC buffer. The membrane was removed from the apparatus, rinsed twice with SSC, air-dried, baked under vacuum at 80 °C for 2 h, and blocked with 5% skim milk in PBS. For lesion detection, the membrane was incubated with anti-(6-4)PP (1:2,000) or anti-CPD antibodies (1:3,000) overnight at 4 °C and then incubated with anti-goat IgG mouse antibody (1:2,500 for (6-4) PPs or 1:5,000 for CPDs) for 1 h at room temperature. The blot was imaged using an ECL system, and the total amount of DNA loaded on the membrane was visualized using SYBR^TM^ Gold staining (Thermo Fisher Scientific).

### Clonogenic survival assay

A total of 500 to 2000 cells were seeded in 60 mm culture plates. The following day, the cells were irradiated with 0, 1, 2, 4, or 8 J/m^2^ of UV-C. Cells were washed and allowed to grow for up to 14 days. Cells were then fixed with 4% PFA for 15 min and stained with 2% of methylene blue in 70% ethanol for 1 to 2 h. After a thorough wash with water, the culture plate was dried and scanned for the quantification. The survival rate was normalized to the number of colonies of untreated cells.

### Flow cytometry

Cells were labeled with 10 mM EdU for 30 min before harvesting and were subsequently processed using the Click-iT^TM^ EdU Flow Cytometry Assay Kit (C10420, Thermo Fisher Scientific) according to the manufacturer’s instructions. In brief, the cells were permeabilized and subjected to a click reaction between EdU and Alexa Fluor 647-picoyl azide. Cells were washed with PBS and then incubated with RNase A (0.1 mg/mL) at 37 °C for 1 h. DNA staining was performed using 0.05 mg/mL propidium iodide as the final concentration. Flow cytometry was conducted using a FACSVerse™ flow cytometer with BD FACSuite™ software (BD Biosciences). Data analysis was performed using the FlowJo software (BD Biosciences).

### Protein extraction and Western blot

For isolation of whole cell extracts, cells were lysed with RIPA buffer (150 mM NaCl, 50 mM Tris-HCl (pH 8.0), 5 mM EDTA, 1% Triton X-100, 0.1% SDS, 0.05 g/mL sodium deoxycholate) supplemented with 1 mM phenylmethylsulfonyl fluoride (PMSF), cOmplete™, EDTA-free protease inhibitor (protease inhibitor, Roche), PhosSTOP™ phosphatase inhibitor (phosphatase inhibitor, Roche), Benzonase® nuclease (Enzynomics) and 5 mM MgCl_2_. For isolation of the chromatin-bound protein fraction, the soluble fraction was isolated by incubating cells in buffer A (100 mM NaCl, 300 mM sucrose, 3 mM MgCl_2_, 10 mM PIPES, pH 6.8, 1 mM EGTA, 0.2% Triton X-100™, phosphatase inhibitor, and protease inhibitor) for 5-8 min on ice, followed by PBS wash and centrifugations. After removal of the supernatant, the chromatin-bound fraction was isolated by resuspending the pellet in RIPA buffer supplemented with 1 mM PMSF, protease inhibitor, phosphatase inhibitor, Benzonase® nuclease and 5 mM MgCl_2_ for 45 min on ice, followed by sonication and centrifugation. Protein concentration was measured by the Bradford assay (Thermo Fisher Scientific). Protein samples were separated in 4-12% NuPAGE Bis-Tris gels (Thermo Fisher Scientific) or hand-cast 8-12% polyacrylamide gels, transferred to nitrocellulose membrane (Amersham 0.2 µm NC membrane, GE) at 100 V for 2 h with a mini-protein tetra system (Bio-Rad) at 4 °C, and blocked with 5% skim milk in Tris-buffered saline containing 0.1% Tween-20 (TBST) for 1 h at room temperature. Primary antibodies were added to the TBST and incubated overnight at 4 °C. The blot was then washed and incubated with HRP-conjugated Goat anti-rabbit IgG (1:5000) or HRP-conjugated Goat anti-mouse IgG (1:5000) antibodies for 1 h at room temperature and the bands were visualized using chemiluminescent substrate (cat #: 34577, Thermo Fisher Scientific) and ChemiDoc imaging system (Bio-Rad).

### Statistical analysis

Prism 9 (GraphPad Software) was used to generate graphs and analyze data. Error bars represent the standard error of the mean (SEM). For statistics, we used one-way ANOVA or two-tailed unpaired Student’s t-test, as descripted in the figure legends; ****p < 0.0001, ***p < 0.001, **p < 0.01, *p < 0.05 and n.s: not significant. Statistical parameters are described in the figures. Sample size was determined based on prior published studies and feasibility.

## Supplementary information

Supplementry figure

Original western blots

## Data Availability

The mass spectrometry proteomics data have been deposited in the PRIDE partner repository of the ProteomeXchange Consortium with the dataset identifier PXD061212. All the other data are available in the main text or the supplementary materials.
