# Supplementary material for: Proteomic discovery of DEK and NUMA1 as new players in UV-induced DNA damage repair mechanisms
Source: Cell Death Discov. 2025 Nov 24;11:547. doi: 10.1038/s41420-025-02823-z (PMC12644609; doi:10.1038/s41420-025-02823-z)
Supplement: Supplementary file 1 — Supplementry figure [file 41420_2025_2823_MOESM1_ESM.pdf]

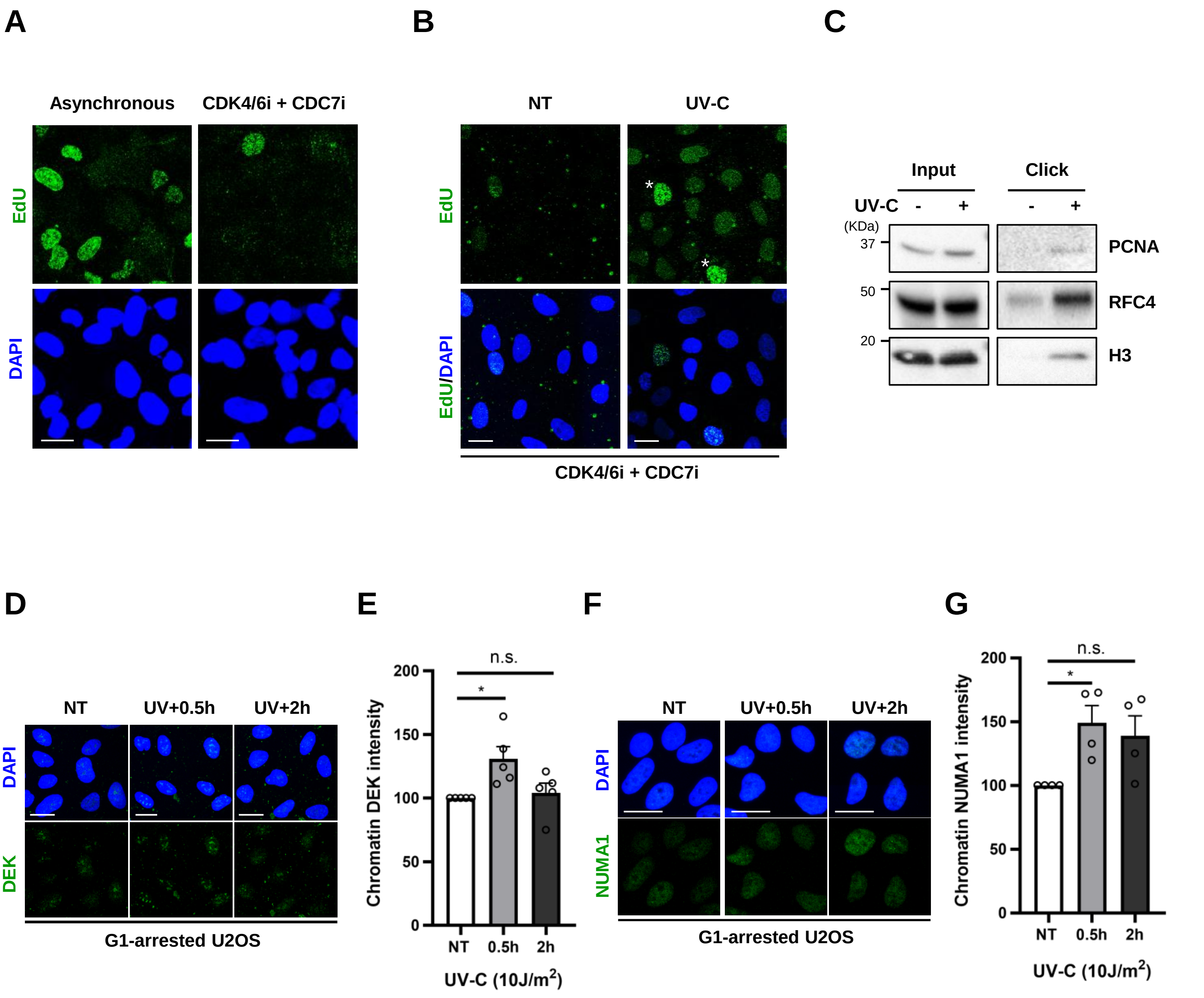

Supplementary Fig. 1. DEK and NUMA1 association with chromatin is increased after UV-C irradiation. (related to Fig. 1)

(A, B) U2OS cells were arrested in G1 phase by treatment with CDK4/6i and CDC7i, incorporated with EdU, and fixed for a click reaction with Alexa Fluor 488 picolyl azide. (B) Cells were irradiated with 30 J/m^2 UV-C before EdU incorporation. NT, no treatment. (C) U2OS cells were treated as shown in Fig 1. a, and proteins eluted by biotin-streptavidin pull-down were subjected to a Western blot. (D-G) U2OS cells were arrested in G1 phase by treatment with CDK4/6i, irradiated with 10 J/m^2 of UV-C, and incubated for the indicated time. Cells were then pre-extracted with CSK buffer, washed, and fixed for DEK (D, E) or NUMA1 (F, G) immunostaining. (D, F) Representative images. (E, G) Quantification of mean fluorescence intensity. Error bars represent SEM (n=4 (E), n=5 (G)). Statistical analysis: one-way ANOVA (E, G). *$P < 0.05$, ns, not significant. (A, B, D, F) Scale bars, 20 μm.

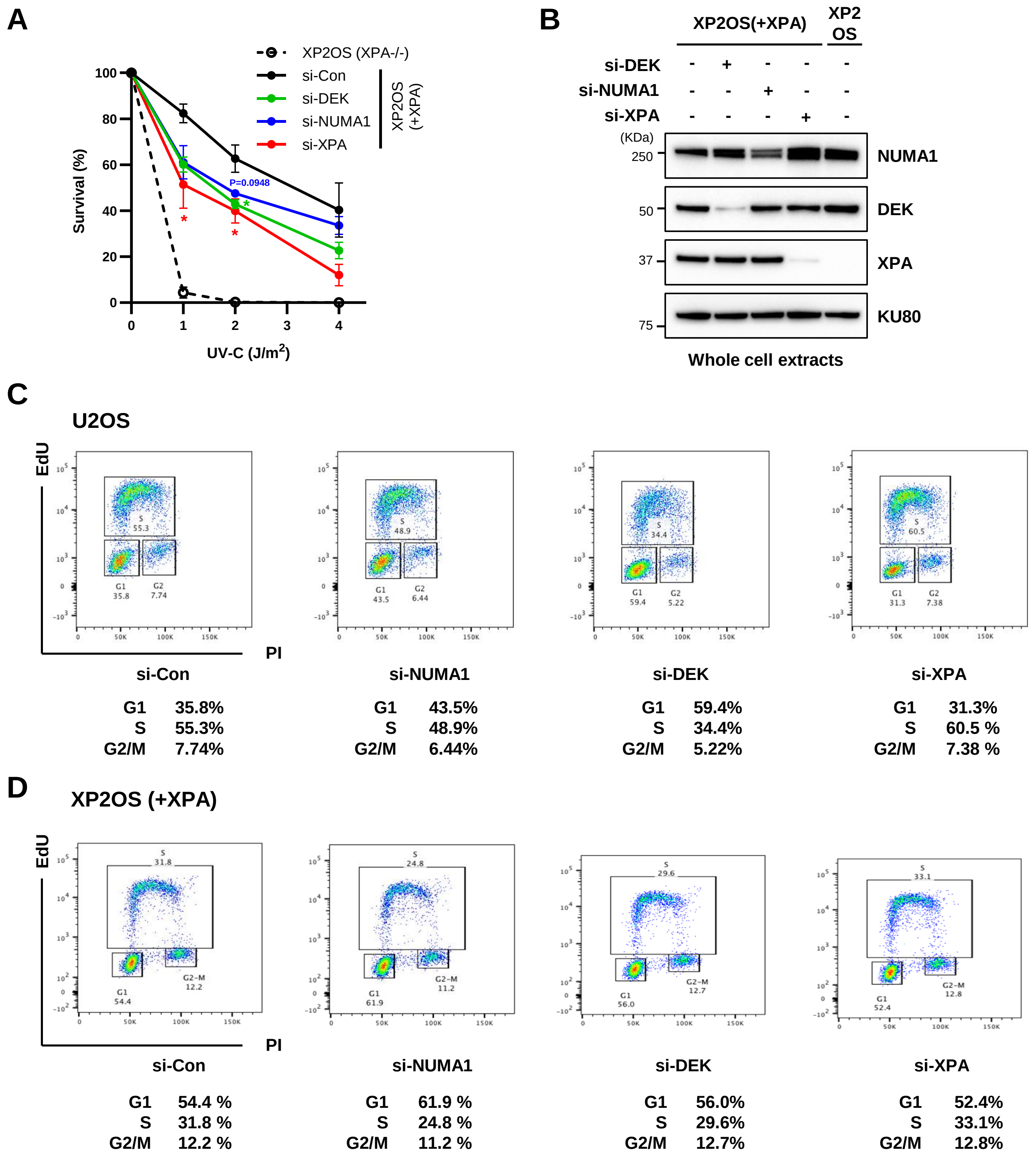

Supplementary Fig. 2. Clonogenic survival assay and the cell cycle profile of DEK- or NUMA1-depleted cells. (related to Fig. 1)

(A, B) XP2OS and XP2OS (+XPA) cells were transfected with siRNAs as indicated. 48 h after transfection, cells were irradiated with UV-C and subjected to clonogenic survival assay (A) or lysed for Western blot (B). (A) Error bars represent standard error of the mean (SEM) (n>3). Each colored asterisk represents the statistical analysis in comparison to si-Con. Statistical analysis: one-way ANOVA . *P < 0.05, and otherwise not significant. (C, D) U2OS (C) and XP2OS (+XPA) cells (D) were transfected with *DEK, NUMA1* or *XPA* siRNAs. 48 h after transfection, cells were incorporated with EdU and fixed for flow cytometry. EdU incorporation was monitored by a click reaction with Alexa Fluor 647 picolyl azide and the DNA content was determined by propidium iodide (PI) staining.

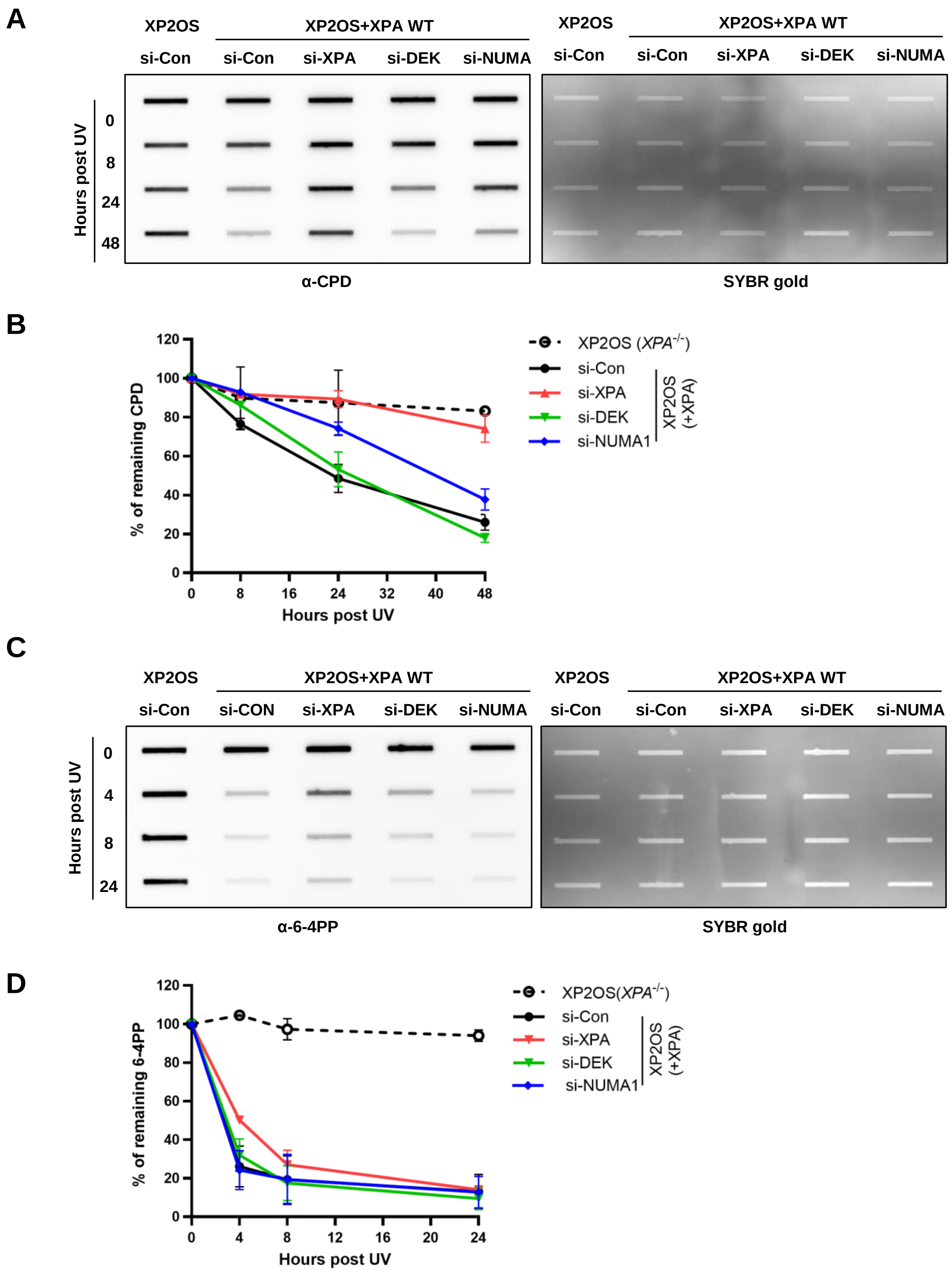

Supplementary Fig. 3. Depletion of DEK or NUMA1 resulted in slight defects in UV lesion repair in slot blot assay. (related to Fig. 2)

(A-D) XP2OS and XP2OS (+XPA) cells were transfected with *DEK*, *NUMA1* or *XPA* siRNAs. 48 h after transfection, the cells were irradiated with 5 J/m^2 of UV-C and collected at the indicated time points, and genomic DNA was isolated for the slot blot assay for CPD (A,B) and (6-4)PPs (C, D) detection. (A, C) Representative images of the slot blot assay. (B, D) Quantification of band intensity. Band intensities were normalized to the control band at 0 h. Error bar represents SEM (n=2).

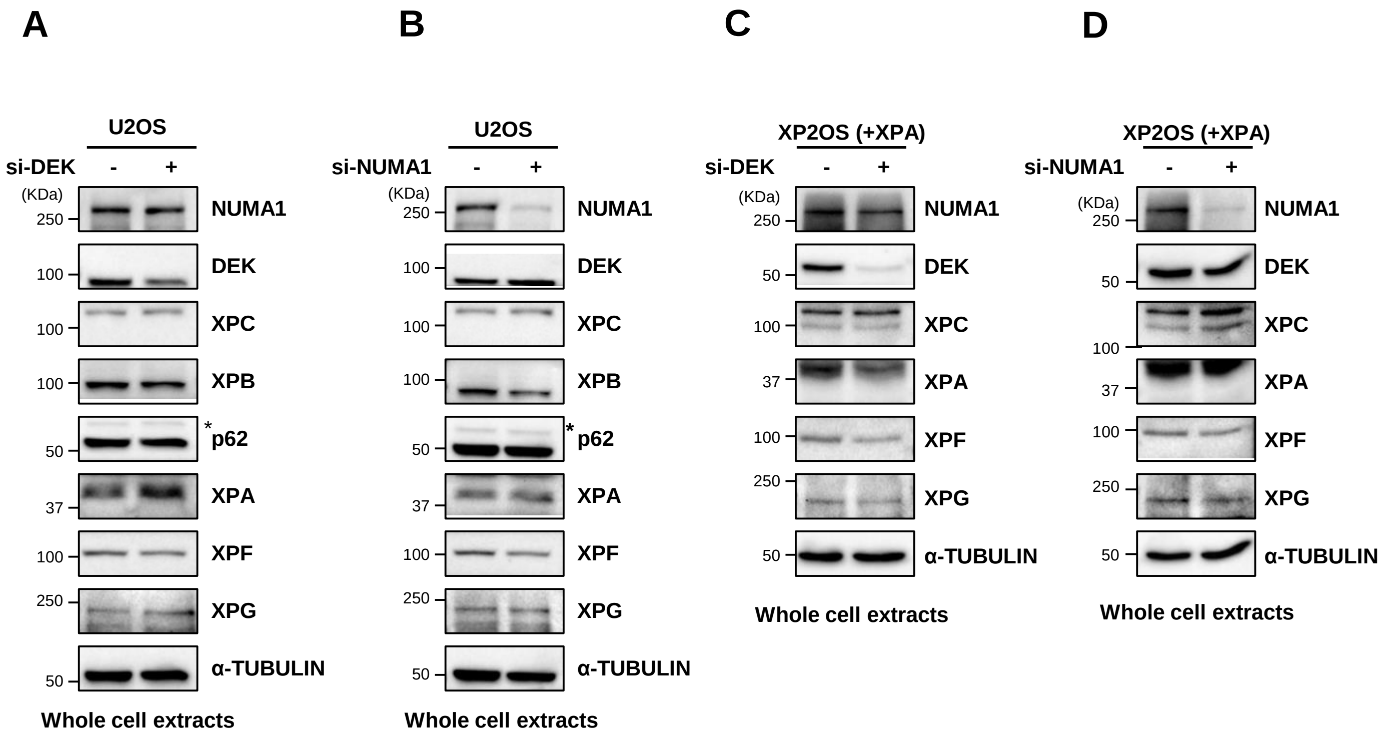

Supplementary Fig. 4. Depletion of DEK or NUMA1 did not affect the protein levels of XP proteins.

(A-D) U2OS cells (A, B) and XP2OS (+XPA) (C, D) cells were transfected with *DEK* (A, C) or *NUMA1* (B, D) siRNAs. 48 h after transfection, whole-cell protein extracts were prepared for a Western blot. (A, B) Asterisk: non-specific band.

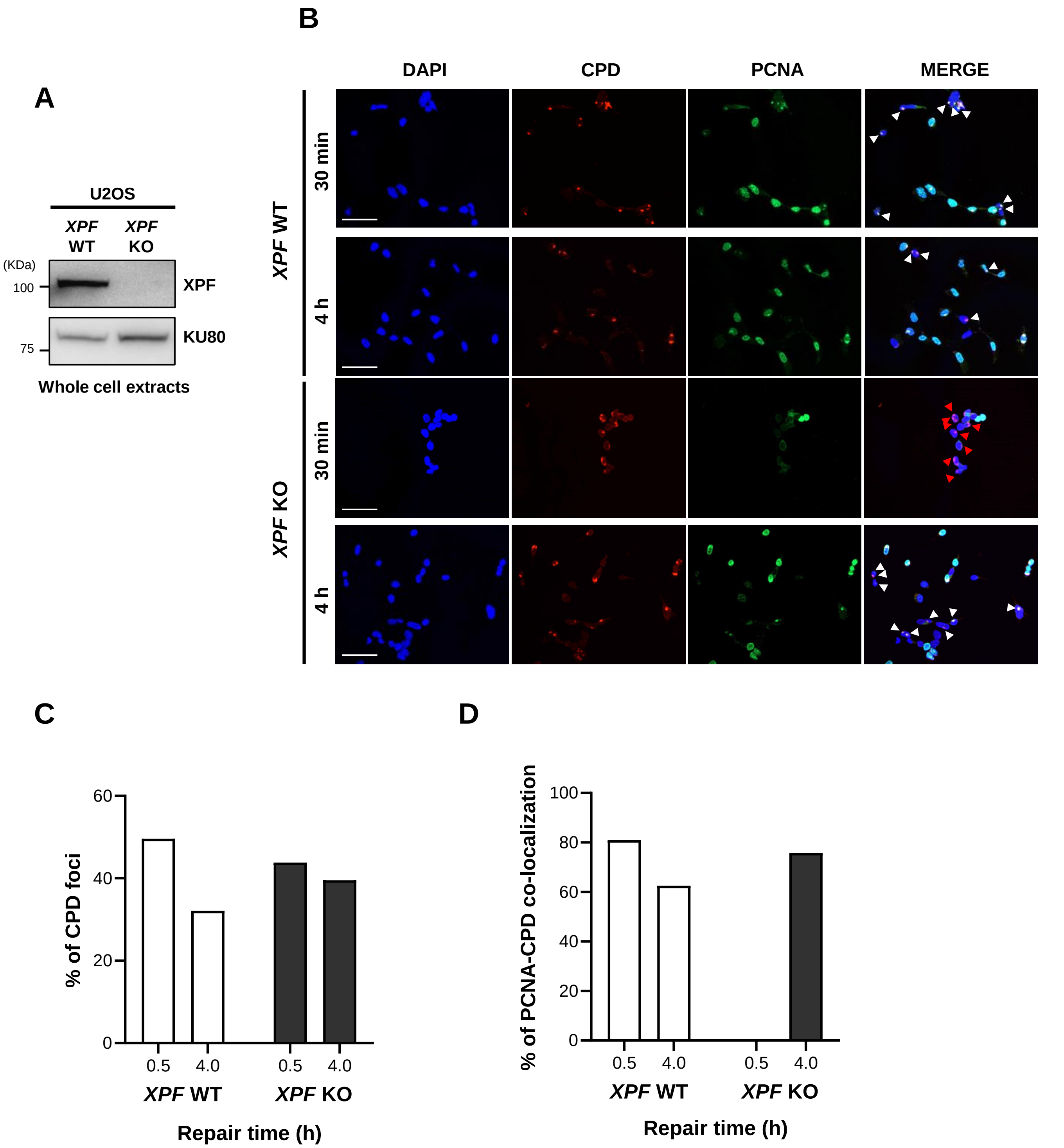

Supplementary Fig. 5. PCNA was recruited to UV lesions in the absence of XPF. (related to Fig. 6)

(A) *XPF* wild-type (WT) and KO U2OS cells were lysed for Western blot analysis. (B-D) *XPF* WT and KO U2OS cells were irradiated through 5 µm micropore filter with 100 J/m^2 of UV-C and fixed at the indicated time points. (B) Representative images of PCNA co-localization with CPDs. Red arrowheads indicate CPDs not colocalized with PCNA and white arrowheads indicate CPDs colocalized with PCNA in non-S phase. Scale bar, 50 µm. (C, D) Quantification of CPD-positive nuclei (%) (n=1) (C) and PCNA co-localization with CPDs (%) (n=1) (D). S phase cells with strong PCNA signal were excluded from the analysis.

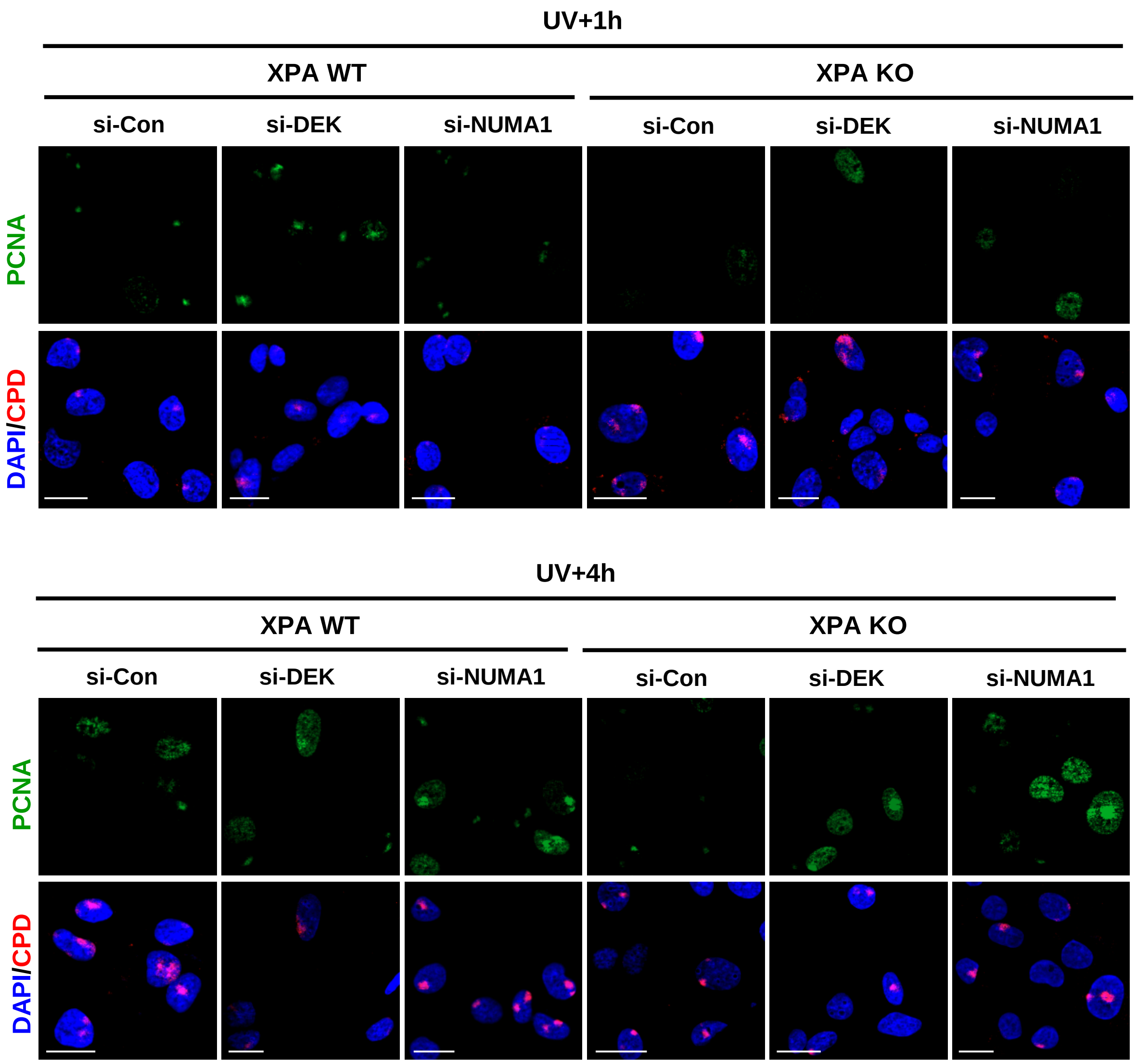

Supplementary Fig. 6. PCNA was recruited to UV lesions at 4h after irradiation in the absence of XPA. (related to Fig. 6A and 6B)

Representative images of PCNA co-localization with CPDs in *XPA* wild-type (WT) and KO U2OS cells, which were transfected with indicated siRNAs, irradiated through 5 μm micropore filter with 100 J/m^2 of UV-C and fixed at the indicated time points. Scale bar, 20 μm.
