# Supplementary figures and images for: Proteomic discovery of DEK and NUMA1 as new players in UV-induced DNA damage repair mechanisms
Source: Cell Death Discov. 2025 Nov 24;11:547. doi: 10.1038/s41420-025-02823-z (PMC12644609; doi:10.1038/s41420-025-02823-z)

Fig.1H

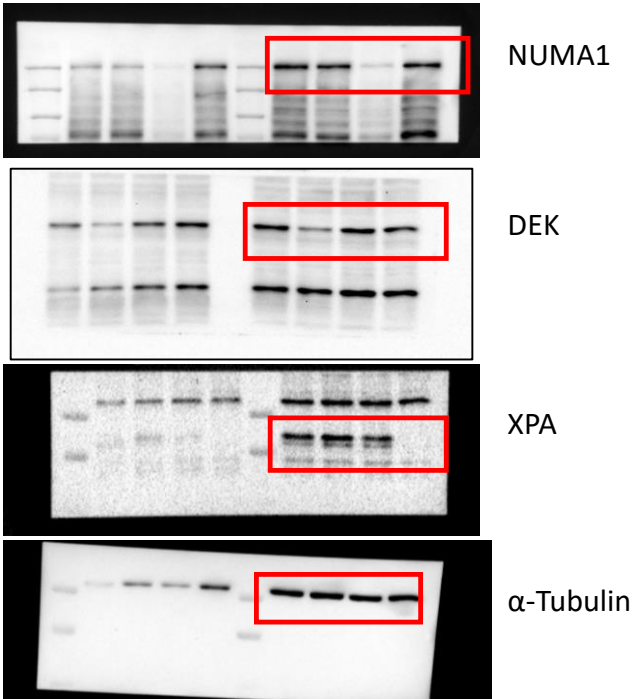

Fig.1J

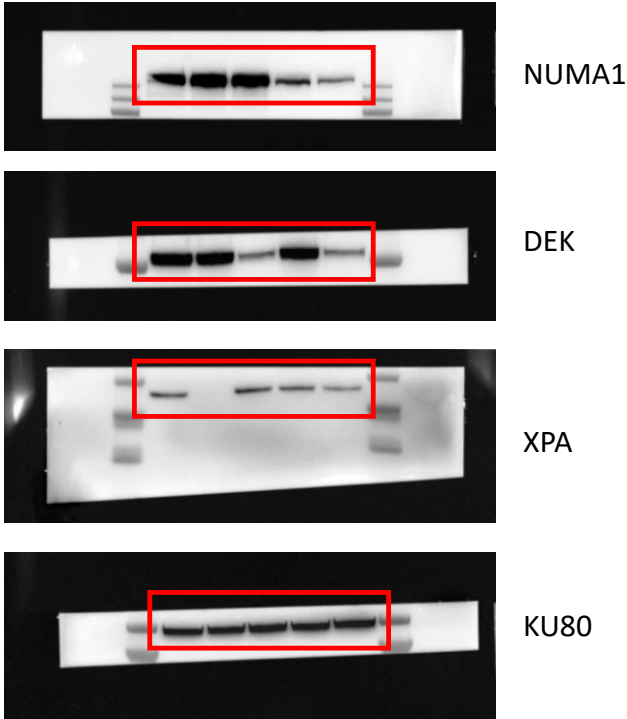

Fig.5G

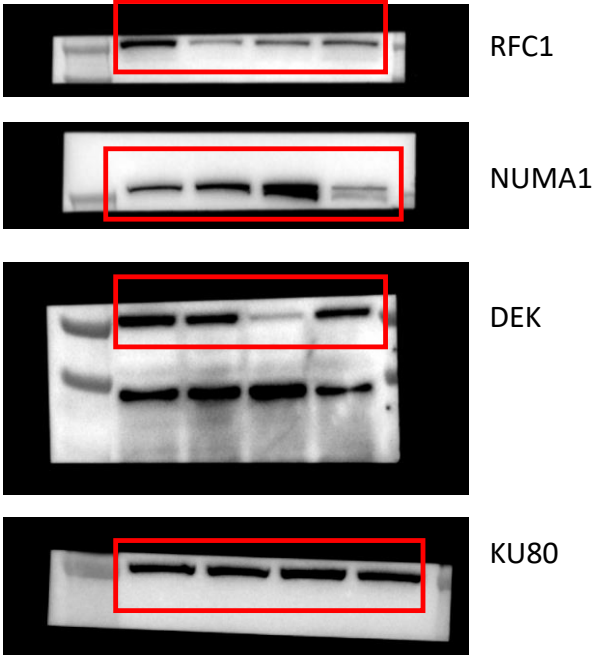

Fig.6C

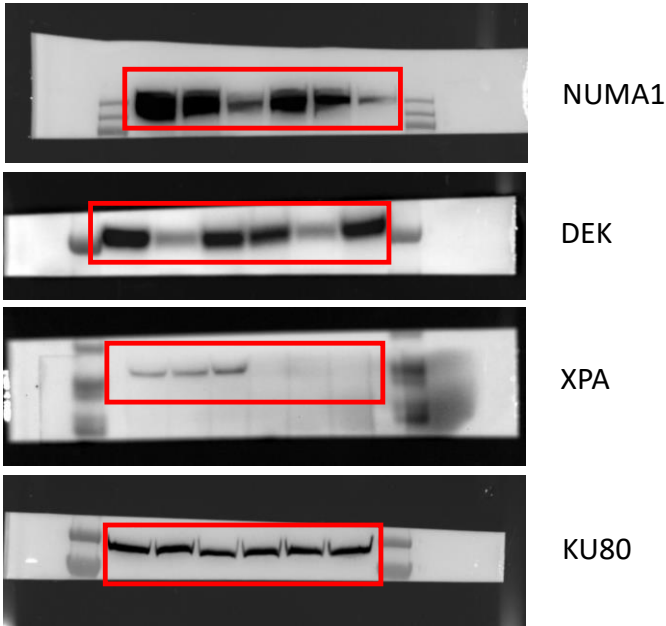

Fig.6E

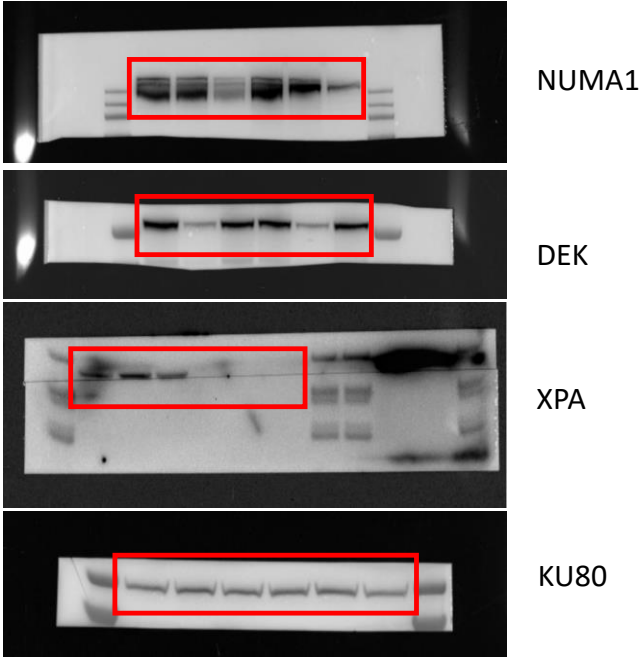

Fig.7A

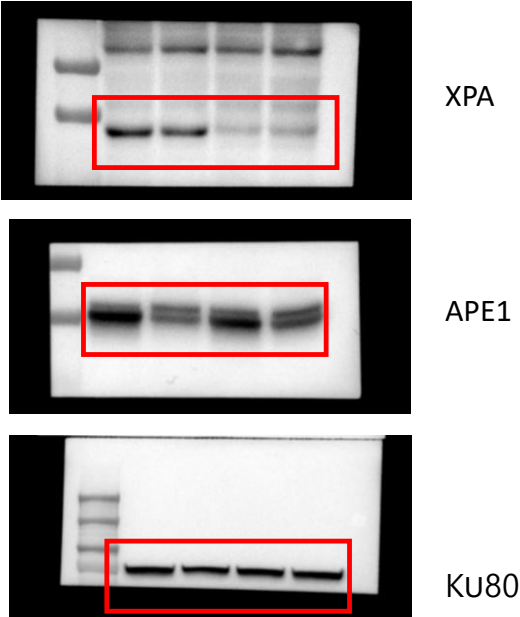

Fig.7E

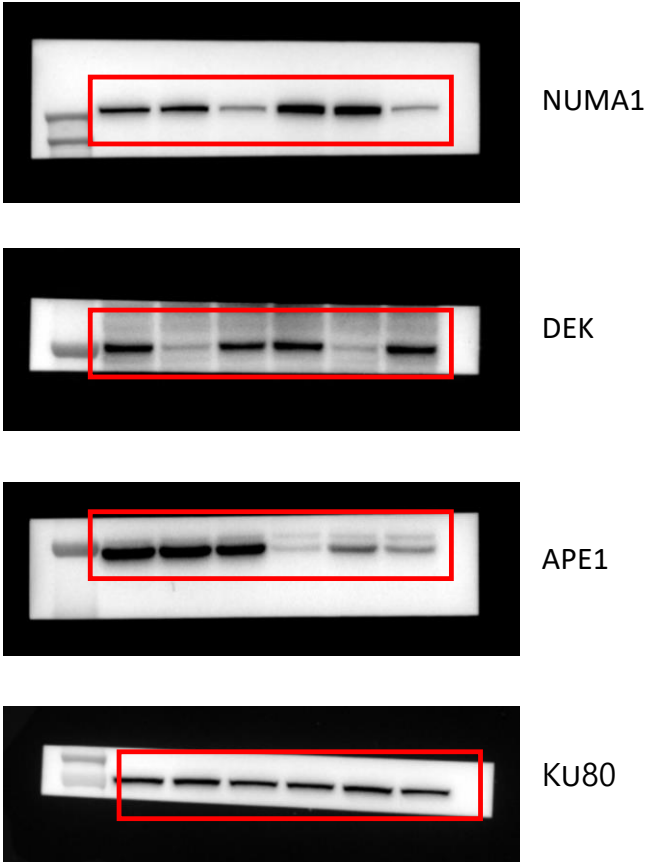

**Fig.S1C**

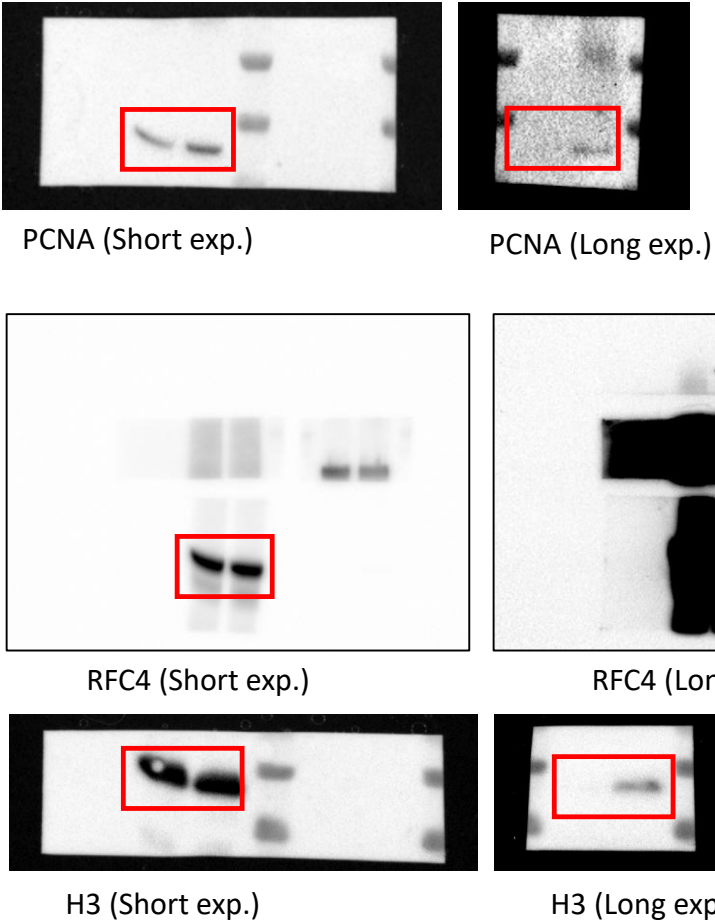

**Fig.S2B**

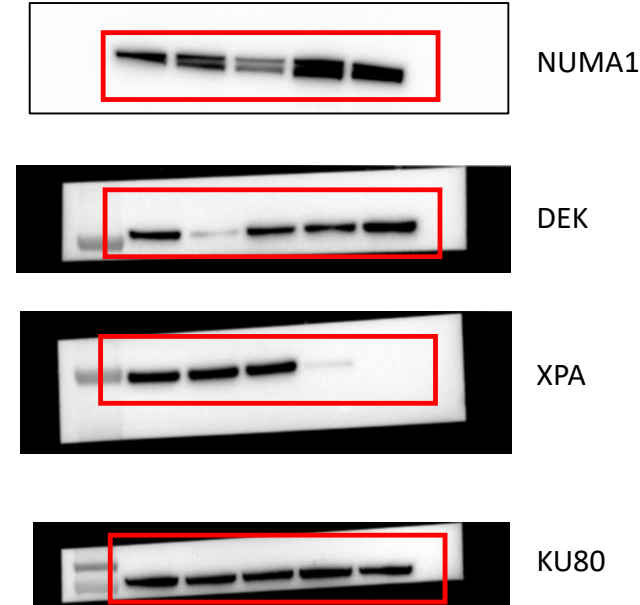

**Fig.S4A**

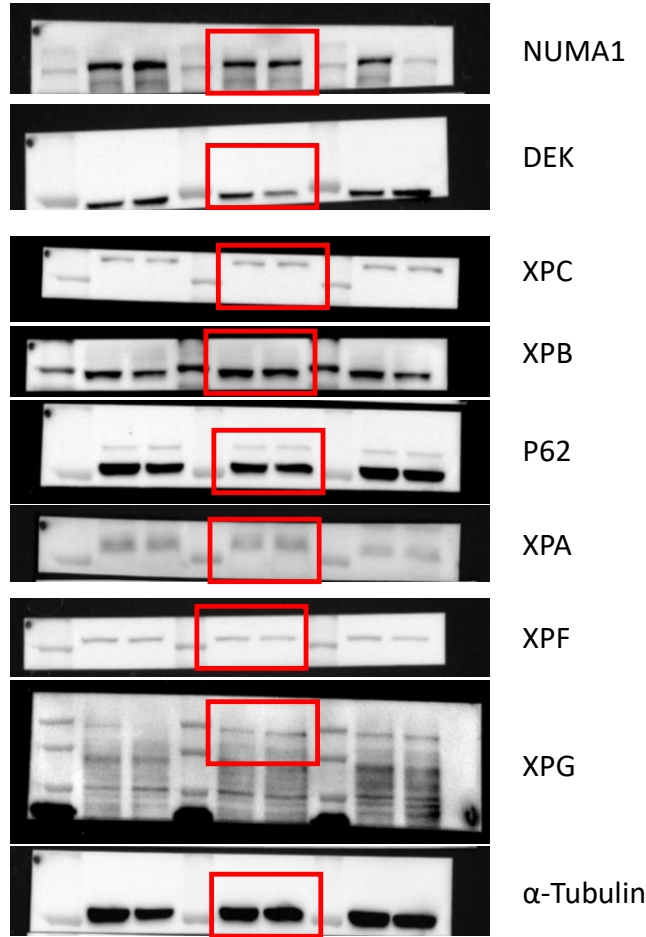

**Fig.S4B**

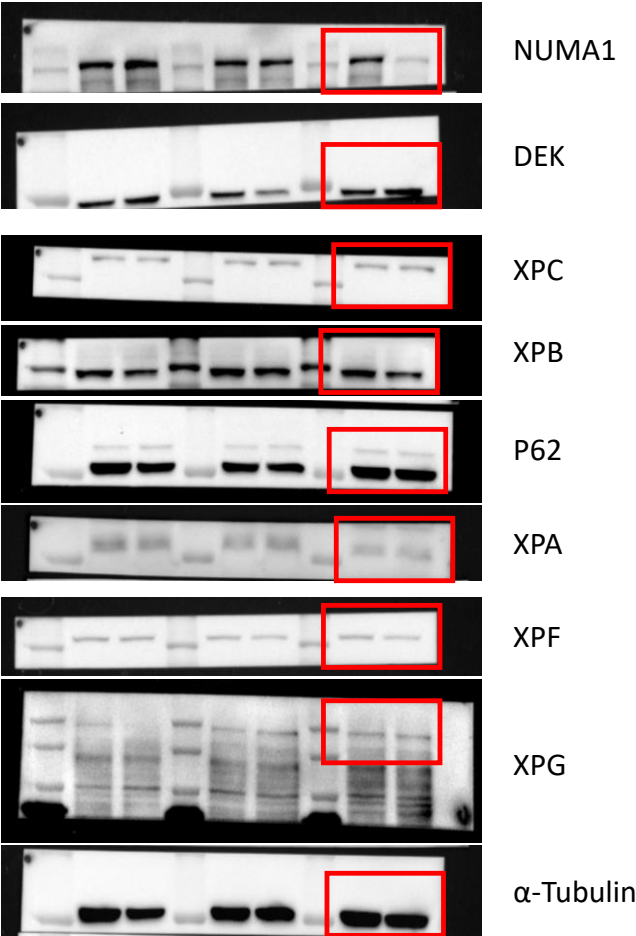

**Fig.S4C**

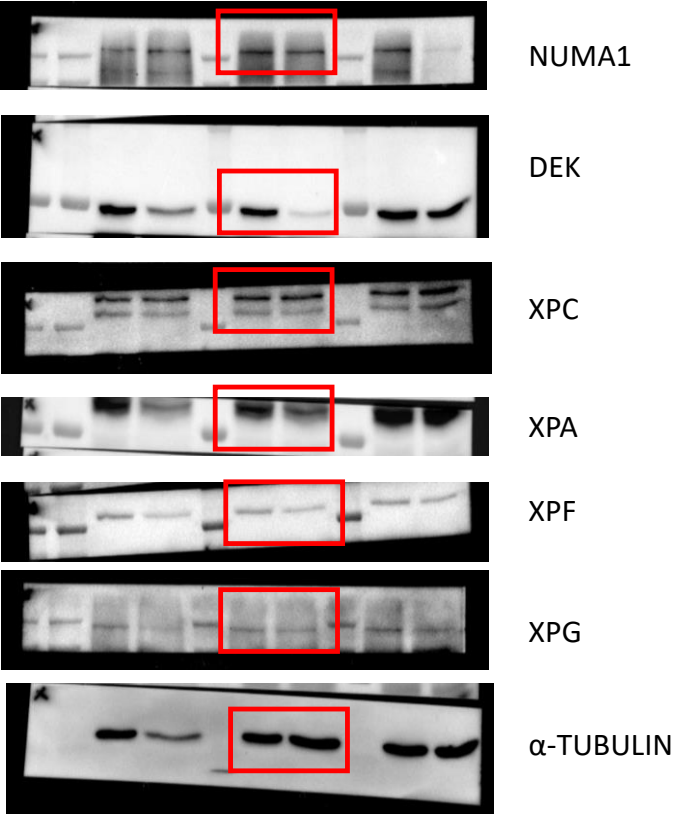

**Fig.S4D**

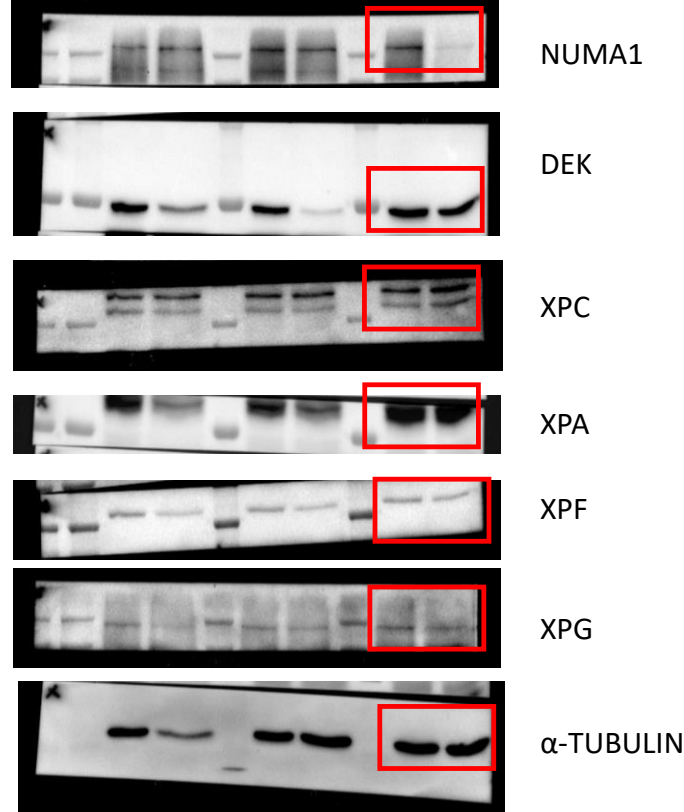

**Fig.S5A**

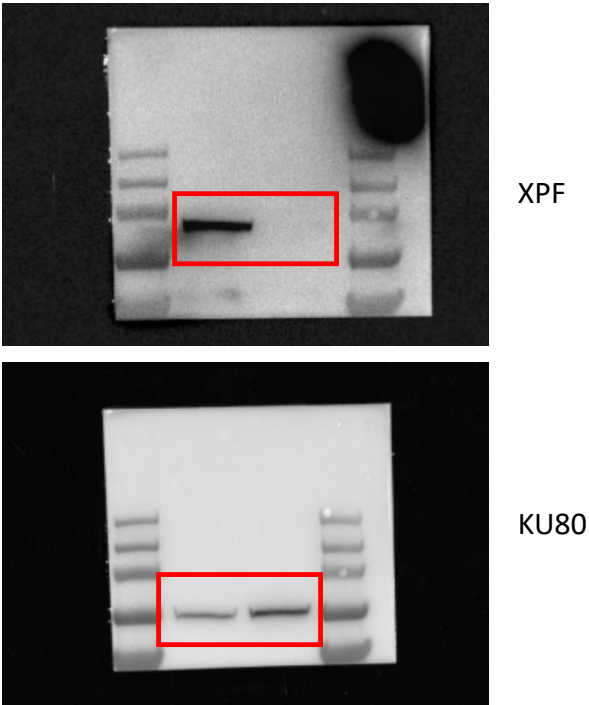

Supplement: Supplementary file 2 — Original western blots [file 41420_2025_2823_MOESM2_ESM.pdf]
